# Prenylated indole-terpenoids with antidiabetic activities from *Penicillium* sp. HFF16 from the rhizosphere soil of *Cynanchum bungei* Decne

**DOI:** 10.3389/fmicb.2023.1099103

**Published:** 2023-03-02

**Authors:** Xijin Liu, Fandong Kong, Na Xiao, Xiaoyu Li, Mingyu Zhang, Fujin Lv, Xiaolin Liu, Xiangchuan Kong, Jing Bi, Xinyi Lu, Daqing Kong, Gangping Hao, Liman Zhou, Guojun Pan

**Affiliations:** ^1^College of Life Sciences, Shandong First Medical University & Shandong Academy of Medical Sciences, Tai'an, Shandong, China; ^2^Key Laboratory of Chemistry and Engineering of Forest Products, State Ethnic Affairs Commission, Guangxi Key Laboratory of Chemistry and Engineering of Forest Products, Guangxi Collaborative Innovation Center for Chemistry and Engineering of Forest Products, School of Chemistry and Chemical Engineering, Guangxi Minzu University, Nanning, China; ^3^State Key Laboratory of Crop Biology, College of Agronomy, Shandong Agriculture University, Tai'an, Shandong, China

**Keywords:** fungus, *Penicillium* sp. HFF16, indole-terpenoids, Antidiabetic activity, *Cynanchum bungei* Decne

## Abstract

Finding novel and effective suppression of hepatic glucagon response antidiabetic compounds is urgently required for the development of new drugs against diabetes. Fungi are well known for their ability to produce new bioactive secondary metabolites. In this study, four new prenylated indole-terpenoids (**1-4**), named encindolenes I-L, as well as a known analogue (**5**), were isolated from the fungus *Penicillium* sp. HFF16from the rhizosphere soil of *Cynanchum bungei* Decne. The structures of the compounds were elucidated by spectroscopic data and ECD analysis. In the antidiabetic activity assay, compounds **1-5** could inhibit glucagon-induced hepatic glucose output with EC_50_ values of 67.23, 102.1, 49.46, 25.20, and 35.96 μM, respectively, and decrease the intracellular cAMP contents in primary hepatocytes.

## Introduction

**Figure 1:**
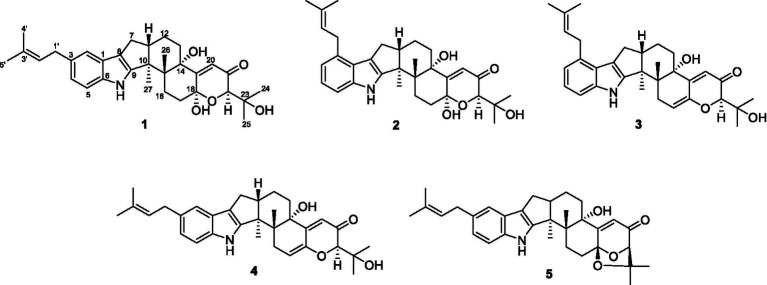
The chemical structures of compounds **1**-**5**.

The liver plays a major role in whole body glucose metabolism by maintaining a balance between glucose production and glucose storage (Lewis et al., 2021; Zhang et al., 2022). Excessive hepatic glucose production contributes substantially to diabetes, and it is proposed that suppression of hepatic glucose production may provide therapeutic advantages for the control of diabetes (Xiao et al., 2017; Liao et al., 2021). During fasting, hepatic gluconeogenesis is the primary source of glucagon-mediated endogenous glucose production (Unger and Cherrington, 2012). Glucagon, a pancreas-derived hormone induced by fasting, promotes gluconeogenesis through induction of intracellular cAMP production. Glucagon promotes hepatic gluconeogenesis through upregulation of cAMP/PKA signaling pathway and prevents hypoglycemia (Zhang et al., 2019). Therefore, finding novel and effective inhibition of glucagon-mediated gluconeogenesis bioactive compounds are urgently required. Fungal secondary metabolites have been proven to be an important source of natural compounds with novel structures and unique activities, many of which contribute to drug discovery and are approved by the US Food and Drug Administration (Pan G. J. et al., 2021; Shankar and Sharma, 2022). The paxilline-type indole-diterpenoids are one of the largest classes of fungal indole-terpenoids (Kong et al., 2019), many of which have significant bioactivities. In our preliminary search for bioactive metabolites from *Penicillium* sp. HFF16, from the rhizosphere soil of *Cynanchum bungei* Decne from Mount Tai, China, nine new indole-terpenoids with weak anti-inflammatory activities and antidiabetic effects were investigated (Pan G. et al., 2021; Xiao et al., 2022). Considering such a significant work, *Penicillium*sp.HFF16 was re-fermented and chemical investigation on its extracts revealed another four new indole-terpenoids (**1**-**4**) (Figure 1). All of the compounds exhibited moderate antidiabetic effects on glucagon-stimulated cAMP accumulation and hepatic glucose output in primary hepatocytes. Herein, the isolation, structural elucidation, and bioactivities of these compounds were described.

## Materials and methods

### General experimental procedures

Optical rotations were measured on an Anton PaarMCP-100 digital polarimeter, and UV spectra were measured on a Beckman DU 640 spectrophotometer. ECD data were collected using a JASCO J-715 spectropolarimeter. NMR spectra were recorded on a Bruckmercury Plus-400 spectrometers with TMS as an internal standard. HRESIMS spectra were recorded with a Micromass Autospec-Uitima-TOF. Infrared (IR) spectra were obtained on a FTIR-650 spectrometer. Semi-preparative high-performance liquid chromatography (HPLC) was carried out using an ODS column (YMC-pack ODS-A, 10 × 250 mm, 5 μm, 4 mL/min). Thin-layer chromatography (TLC) and column chromatography (CC) were performed on plates precoated with silica gel GF_254_ (10–40 μm, Yantai Jiangyou Silicone Development Co. Ltd).

### Fungal material and fermentation

The fungus *Penicillium* sp. HFF16 was isolated from the rhizosphere soil of *Cynanchum bungei* Decne, in Mount Tai, China in May 2020 and identified according to its morphological characteristics and ITS gene sequences (Pan G. et al., 2021). A reference culture of *Penicillium* sp. HFF16 maintained at -80°C is deposited in our laboratory. The isolate was cultured on the plates of PDA medium at 28°C for 4 days. Plugs of agar supporting mycelium growth were cut and transferred aseptically to the 10 × 250-mL Erlenmeyer flasks each containing 100 mL of liquid medium (potato 200 g, glucose 20 g per liter of tap water) and cultured at 28°C at 150 RPM for 3 days. The seed liquid was inoculated aseptically into the 200 × 1,000-mL Erlenmeyer flasks each containing rice medium (80 g rice, 100 mL of tap water) at 0.5–1% inoculation amount and incubated at room temperature under static conditions for 35 days.

### Extraction and isolation

The cultures (16 kg) were then extracted into 50 L of ethyl acetate (EtOAc) by soaking overnight. The extraction repeated for three times. The combined EtOAc extracts were dried under vacuum to produce 52.1 g of extract. The EtOAc extract was subjected to a silica gel column-vacuum liquid chromatography column, eluting with a stepwise gradient of 0, 9, 11, 15, 20, 30, 50, and 100% EtOAc in petroleum ether (v/v), to give seven fractions (Fr. 1-7). Fraction 2 (17.2 g) was applied to ODS silica gel with gradient elution of CH_3_OH (MeOH)-H_2_O (1:5, 2:3, 3:2, 4:1, 1:0) to yield four subfractions (Fr. 2-1-Fr. 2-4). Fr. 2-1 (5.0 g) was applied to ODS silica gel with gradient elution of MeCN-H_2_O (1,4, 2:3, 3:2, 7:3, 4:1, 9:1, 9.5:1, and 0) to yield six tertiary fractions (Fr. 2-1-1-Fr. 2-1-6). Fr. 2-1-3 (0.81 g) was purified using semi-prep HPLC (isocratic system 93.6% MeOH/H_2_O, v/v) to yield nine fourthiary fractions (Fr. 2-1-3-1-Fr. 2-1-3-9). Fr. 2-1-3-8 (49 mg) was purified using semi-prep HPLC (isocratic system 90% MeCN/H_2_O, v/v) to give compounds **1** (*t*_R_10.1 min; 10 mg), **4** (*t*_R_6.1 min; 11 mg), and **5** (*t*_R_ 8.1 min; 13 mg). Fr. 2-1-3-7 (62 mg) was purified using semi-prep HPLC (isocratic system 90% MeCN/H_2_O, v/v) to give compounds **3** (*t*_R_5.8 min; 9 mg) and **2** (*t*_R_9.9 min; 11 mg).

*Encindolene I (**1**)*: white powder; [*α*]25 D-10 (*c* 0.1, MeOH); UV (MeOH) *λ*_max_ (log *ε*): 288 (2.96), 233 (3.47) nm;IR (KBr) *ν*_max_: 3391, 2,961, 2,923, 1,667, 1,453, 1,260, 1,024 cm^−1^; ECD (MeOH) *λ*_max_ 205 (−13.71), 229 (+4.43), 260 (+4.57), 325 (+6.60) nm. ^1^H and ^13^C NMR data, Tables 1, 2; HRESIMS *m/z*542.2862 [M + Na]^+^ (calcd for C_32_H_41_NO_5_Na, 542.2877).

**Table 1:** The ^13^C NMR (100 MHz) data of compounds, **1-4** in CD_3_OD.

Position	1	2	3	4
	*δ*_C_			
1	125.7, C	124.8, C	124.8, C	125.7, C
2	117.2, CH	132.7, C	132.7, C	117.2, CH
3	132.3, C	118.4, CH	118.4, CH	132.3, C
4	120.9, CH	120.3, CH	120.3, CH	120.9, CH
5	111.6, CH	109.7, CH	109.8, CH	111.6, CH
6	139.5, C	140.9, C	141.1, C	139.7, C
7	27.3, CH_2_	29.4, CH_2_	29.4, CH_2_	27.3, CH_2_
8	116.1, C	115.8, C	115.8, C	116.0, C
9	153.1, C	152.2, C	152.0, C	152.9, C
10	51.3, C	51.1, C	50.9, C	51.2, C
11	50.2, CH	50.5, CH	50.2, CH	50.0, CH
12	21.5, CH_2_	21.5, CH_2_	21.6, CH_2_	21.7, CH_2_
13	33.5, CH_2_	33.7, CH_2_	32.7, CH_2_	32.7, CH_2_
14	77.2, C	77.2, C	75.3, C	75.3, C
15	43.4, C	43.5, C	43.5, C	43.5, C
16	26.0, CH_2_	26.0, CH_2_	31.5, CH_2_	31.5, CH_2_
17	28.1, CH_2_	28.1, CH_2_	112.7, CH	112.7, CH
18	98.4, C	98.4, C	145.7, C	145.7, C
19	160.6, C	160.7, C	156.0, C	156.0, C
20	122.1, CH	122.1, CH	116.3, CH	116.3, CH
21	198.4, C	198.3, C	196.9, C	196.9, C
22	78.5, CH	78.6, CH	86.4, CH	86.4, CH
23	72.4, C	72.3, C	73.9, C	73.8, C
24	24.9, CH_3_	24.9, CH_3_	25.9, CH_3_	25.9, CH_3_
25	25.2, CH_3_	25.2, CH_3_	26.0, CH_3_	26.0, CH_3_
26	19.4, CH_3_	19.3, CH_3_	20.4, CH_3_	20.4, CH_3_
27	15.7, CH_3_	15.6, CH_3_	15.8, CH_3_	15.9, CH_3_
1’	34.8, CH_2_	32.4, CH_2_	32.4, CH_2_	34.7, CH_2_
2’	125.4, CH	124.8, CH	124.8, CH	125.4, CH
3’	131.1, C	131.2, C	131.2, C	131.0, C
4’	25.2, CH_3_	25.2, CH_3_	25.2, CH_3_	25.2, CH_3_
5’	17.1, CH_3_	17.3, CH_3_	17.3, CH_3_	17.1, CH_3_

**Table 2:** The ^1^H NMR (400 MHz) data of compounds, **1-4** in CD_3_OD.

Position	1	2	3	4
	*δ*_H_ (*J* in Hz)			
2	7.09, s			7.07, s
3		6.70, d (7.8)	6.70, d (7.8)	
4	6.81, br d (8.1)	6.87, t (7.8)	6.87, t (7.8)	6.79, br d (8.3)
5	7.14, br d (8.1)	7.10, d (7.8)	7.12, d (7.8)	7.17, d (8.3)
7	2.35, dd (11.6, 11.6)	2.52, overlap	2.53, dd (13.0, 14.6)	2.35, overlap
	2.66, overlap	2.81, overlap	2.82, overlap	2.66, overlap
11	2.70, overlap	2.84, m	2.82, m	2.80, m
12	1.63, m	1.70, m	1.70, m	1.74, m
	2.01, m	2.04, m	2.10, m	2.06, m
13α	1.63, overlap	1.77, overlap	2.07, m	2.07, m
13β	1.60, overlap	1.70, overlap	1.98, m	1.97, m
16α	2.47, m	2.48, m	2.38, dd (17.7, 6.5)	2.38, overlap
16β	1.73, m	1.75, m	3.18, br d (17.7)	3.18, br d (17.7)
17α	2.53, m	2.55, m	5.75, m	5.75, m
17β	1.99, m	1.99, m		
20	5.80, s	5.87, s	5.96, s	5.95, s
22	4.05, s	4.07, s	4.12, s	4.12, s
24	1.31, s	1.32, s	1.32, s	1.32, s
25	1.30, s	1.31, s	1.26, s	1.26, s
26	0.85	1.02	1.09	1.07
27	1.28, s	1.32, s	1.35, s	1.35, s
1’	3.37, d (7.6)	3.57, m	3.56, m	3.36, d (7.6)
2’	5.38, br t (7.6)	5.36, br t (7.2)	5.36, br t (7.2)	5.36, br t (7.6)
4’	1.74, br s	1.74, br s	1.73, br s	1.74, br s
5’	1.75, br s	1.76, br s	1.76, br s	1.74, br s

*Encindolene J (**2**)*: white powder; [*α*]25 D-11 (*c* 0.1, MeOH); UV (MeOH) *λ*_max_ (log *ε*): 283 (3.06), 231 (3.56) nm;IR (KBr) *ν*_max_: 3453, 2,953, 2,921, 1,670, 1,453, 1,375, 1,172 cm^−1^; ECD (MeOH) *λ*_max_ 207 (−26.39), 226 (+5.56), 248 (+10.57), 302 (+2.26) nm. ^1^H and ^13^C NMR data, Tables 1, 2; HRESIMS *m/z*542.2864 [M + Na]^+^ (calcd for C_32_H_41_NO_5_Na, 542.2877).

*Encindolene K (**3**)*: white powder; [*α*]25 D + 354 (*c* 0.1, MeOH); UV (MeOH) *λ*_max_ (log *ε*): 283 (3.06), 231 (3.50) nm;IR (KBr) *ν*_max_: 3427, 2,930, 1,660, 1,373, 1,298, 1,180 cm^−1^; ECD (MeOH) *λ*_max_ 204 (−12.42), 223 (+4.43), 239 (−12.73), 325 (+18.33) nm. ^1^H and ^13^C NMR data, Tables 1, 2; HRESIMS *m/z*502.2947 [M + H]^+^ (calcd for C_32_H_40_NO_4_, 502.2952).

*Encindolene L (**4**)*: white powder; [*α*]25 D + 52 (*c* 0.1, MeOH); UV (MeOH) *λ*_max_ (log *ε*): 288 (2.98), 233 (3.47) nm;IR (KBr) *ν*_max_: 3428, 2,929, 2,923, 1,660, 1,451, 1,297, 1,179 cm^−1^; ECD (MeOH) *λ*_max_ 207 (−8.98), 225 (+6.60), 242 (−10.71), 325 (+16.67) nm. ^1^H and ^13^C NMR data, Tables 1, 2; HRESIMS *m/z*502.2950 [M + H]^+^ (calcd for C_32_H_40_NO_4_, 502.2952).

### Preparation of primary hepatocytes and cell viability assay

Primary hepatocytes were isolated from male C57BL/6 J mice (Jinan Pengyue Experimental Animal Breeding Co. Ltd) by an improved two-step collagenase infusion (Xiao et al., 2017). All experiments and animal care conducted in accordance with the Provision and General Recommendation of Chinese Experimental Animals Administration Legislation and were approved by the Animal Ethics Committee of Shandong Agriculture University. Primary mouse hepatocytes were maintained in DMEM medium with 10% fetal bovine serum (FBS). After attachment, the cells incubated with 100 nM glucagon, as well as the tested compounds. After 24 h, MTT solution was added and incubated for 4 h. The purple crystals were dissolved with dimethylsulfoxide (DMSO) and the absorbance value was determined at 570 nm.

### Hepatic glucose production and intracellular cAMP measurement

Primary hepatocytes on 48-well plates were maintained in DMEM (10% FBS) medium. After attachment, the media was replaced with Krebs-Ringer HEPES buffer to fast the cells for 2 h. Then, the cells were cultured with glucose out media supplemented with 10 mM pyruvate, 100 nM glucagon, or with metformin (1 mM) and the tested compounds (1, 20, 40, 80, and 160 μm). After 6 h, the cell supernatant was collected for glucose analysis. For intracellular cAMP measurement, primary hepatocytes were treated with the tested compounds in the presence or absence of 100 nM glucagon for 4 h. cAMP was calculated in primary hepatocytes with an ELISA kit. All data were expressed as the mean ± SD from at least three independent experiments.

## Results and discussion

### Structure elucidation of compounds

**Figure 2:**
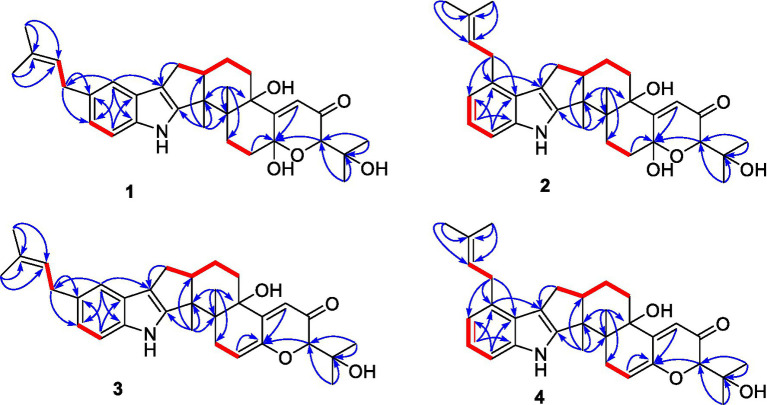
Selected HMBC and COSY correlations of compounds **1**-**4**.

**Figure 3:**
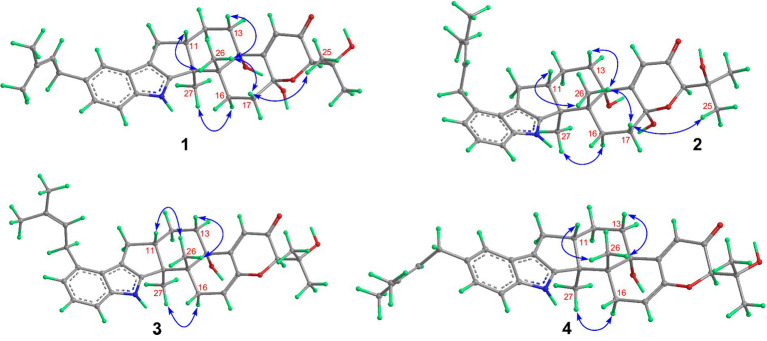
Selected ROESY correlations of compounds **1**-**4**.

**Figure 4:**
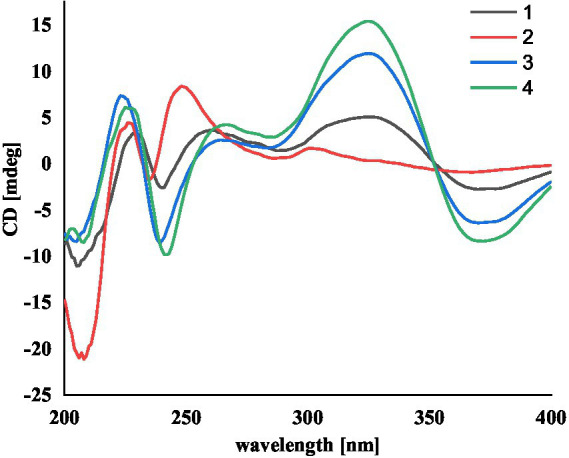
The experimental ECD spectra of compounds **1**-**4**.

Compound **1** was assigned the molecular formula C_32_H_41_NO_5_ by HRESIMS, with 13 degrees of double-bond equivalents. The ^13^C and HSQC NMR spectra (Table 1) of **1** revealed a total of 32 carbons including eight aromatic carbons (three protonated) corresponding to one indole moiety, four olefinic carbons attributed to two double bonds, four oxygenated sp^3^ carbons with one protonated, two sp^3^ quaternary carbons, six sp^3^ methylenes, one sp^3^ non-protonated methine, and six methyls. The presence of a prenyl group was demonstrated by HMBC correlations from the two methyls H_3_-4′ and H_3_-5′ (*δ*_H_ 1.74 and 1.75) to one olefinic quaternary carbon (*δ*_C_ 125.4) and one olefinic methine (*δ*_C_ 131.1) and COSY correlation between the olefinic proton H-2′ (*δ*_H_ 5.38) and the methylene protons H_2_-1′ (*δ*_H_ 3.37). The above data were quite similar to those of the known compound 3-methyl-2-butenylpaspaline (**5**) (Cole et al., 1977), with the main differences being the chemical shifts for the two oxygenated carbons C-18 and C-22, which were *δ*_C_ 98.4 and 78.5 for **1** while 104.4 and 88.0 for **5** (Cole et al., 1977). These data, as well as the less of a H_2_O in the molecule formula compared to that of **5** deduced from the HRESIMS data, suggested that the connection between C-18 and C-22 in **5** was cleaved through hydrolysis to afford **1**. The HMBC and COSY data (Figure 2) further confirmed this deduction. The relative configuration of **1** was assigned by the analysis of its ROESY spectrum (Figure 3). ROESY correlations of H-11/H_3_-26/Hβ-13 suggested the same orientation of these protons and the trans-diaxial relationship of H_3_-26 and OH-14, while correlation of H_3_-27/Hα-16 indicated that these protons located at the face opposite to H_3_-26. ROESY correlations of H_3_-26/Hβ-17/H_3_-25 suggested the same face of these protons, indicating the α orientation of OH-18 and H-22. The experimental ECD spectrum (Figure 4) of **1** showed negative Cotton effects (CEs) around 206, 239, and 373 nm, and positive ones around 228, 220, and 326 nm, respectively (Figure 4), which were very similar to those for encindolenes D and E (Pan G. et al., 2021), two analogs isolated from the same fungus. This led to the assignment of the absolute configurations of **1** as shown in Figure 1.

Compound **2** was obtained as a white powder, and its molecular formula was determined to be the same as that of **1** according to the HRESIMS data, with a molecule of H_2_O less than **1**. The NMR data of **2** were also quite similar to those of **1**. The main differences between the ^1^H NMR spectra of them were that signals attributed to a 1,2,3-trisubstituted benzene ring in **2** replaced those corresponding to a 1,2,6-trisubstituted benzene ring in **1**, indicating the location of the prenyl at C-2 or C-5 in **2**. HMBC correlations from the methylene protons of the prenyl group H_2_-1′ to C-1, C-2, and C-3 in the indole group and COSY correlations of H-3/H-4/H-5 further confirmed this deduction. Their remaining substructures were determined to be identical according to the 2D NMR data. The relative configuration of **2** was deduced to be the same as that of **1** based on their similar NMR chemical shifts. ROESY correlations (Figure 3) of H-11/H_3_-26/Hβ-17/H-24 (25) suggested the face of these protons. ROESY correlation (Figure 3) of H_3_-27/Hα-16 indicated that these protons located at the face opposite to H_3_-26. These data further confirming the above deduction. The absolute configurations of **2** were also assigned as shown in Figure 1 by a comparison of its ECD spectrum with that of **1** (Figure 4), which showed great similarity.

The molecular formula of compound **3** was established as C_32_H_39_NO_4_ by HRESIMS, with one H_2_O less compared to **1** and **2**. The NMR spectra of **3** were closely related to those of **2**, indicating that **3** was also a prenylated indole-diterpenoid. A comparison of the NMR data between **2** and **3** revealed the absence of the dioxygenated non-protonated carbon C-18 and a methylene and the presence of an additional trisubstituted double bond in **2** compared to **3**. COSY correlations of H_2_-16 with the olefinic proton H-17 and HMBC correlation from H_2_-16 to C-17 and C-18 suggested that dehydration occurred at C-17/C-18 in **2** to afford compound **3**. The relative configuration of **3** was proposed to be the same as that of **2** based on a biosynthetic consideration, which was further confirmed by NOESY correlations of H-11/H_3_-26/Hβ-13 and H_3_-27/Hα-16. The ECD spectra of **3** were quite similar to those of **1** and **2** (Figure 4), thus assigning their same absolute configurations for the chiral carbons C-10, C-11, C-14, C-15, C-22, and C-23.

The molecular formula of compound **4** was established to be the same as that of **3** by HRESIMS. Their NMR data were also quite similar. A comparison of the NMR data of **4** with those of **1** revealed that they bear the same 3-prenylated indole moiety. The remaining NMR data of **4** were nearly identical to those of **3**. The above data led to the determination of the structure of **4**, and the only difference between it and **3** was the location of the prenyl group, which was C-2 in **4**. HMBC correlations from H_2_-1′ to C-2, C-3, and C-4, as well as COSY correlations of H-4/H-5, further confirmed this deduction. The relative configuration of **4** was deduced to be the same as that of **3** by their similar NMR data (Tables 1, 2). ROESY correlations of H-11/H_3_-26/Hβ-13 and H_3_-27/Hα-16 further confirmed this deduction. The absolute configuration of **4** was also assigned to be the same as that of **3** by their similar ECD curves (Figure 4).

**SCHEME 1:**
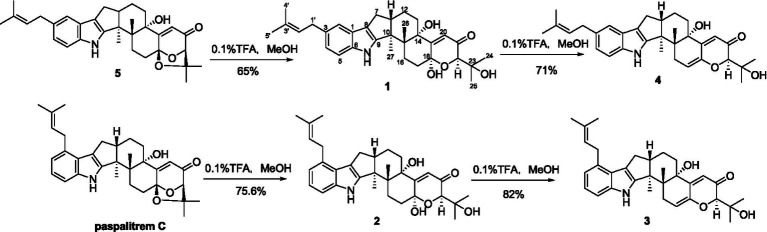
The transformation of **5** to **1** and **4** and paspalitrem C to **2** and **3** in acidic reaction condition.

Compounds **1-5** are structurally closely related. Compounds **1** and **4** could be the dehydration products of **5**, while **2** and **3** could be the dehydration products of another known compound paspalitrem C. Therefore, it is necessary to define whether these compounds were artificial products due to acidic dehydration during the purification process and find the reaction conditions of mutual transformation between them to lay a foundation for the accumulation of these compounds. The experiment of mutual transformation between these compounds was performed. The results indicated that compound **5** could be converted to compounds **1** and **4** in 0.1% trifluoroacetic acid in methanol, and paspalitrem C, a previously isolated analog from the same fungus, can be converted to compounds **2** and **3** under the same conditions (Scheme 1). However, after the treatment of silica gel and C18, there is no structural transformation, but in other strong acids such as hydrochloric acid and sulfuric acid, the compound is basically degraded without effective results. These results indicated that the production of compounds **1**-**4** is probably the result of enzyme catalysis, but it cannot exclude the possibility that compounds **1**-**4** may be artificial products from **5** and paspalitrem C due to the slightly acidic growth environment in the late stage of cultivation. These results also suggested that the absolute configurations of all the chiral carbon except for C-18 in **1**, **2**, and **5** were the same.

**SCHEME 2:**
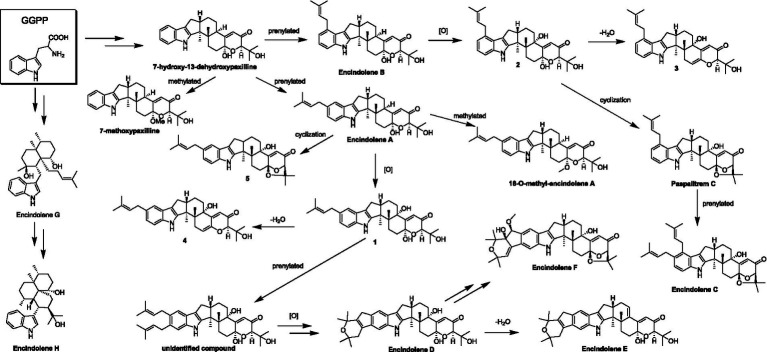
Hypothetical biosynthetic pathway for the skeletons of the indole-terpenoids isolated from *Penicillium sp.* HFF16.

Until now, a total of 17 indole-terpenoids including compounds **1**-**5** and other twelve previously reported analogs such as encindolenes A-C, 18-O-methyl-encindolene A (Pan G. et al., 2021), encindolenes D-H (Xiao et al., 2022), paspalitrem C (Dorner et al., 1984), 7-methoxypaxilline (Ariantari et al., 2019), and 7-hydroxy-13-dehydroxypaxilline (Peter and Christopher, 1994) have been identified from *Penicillium* sp. HFF16, and the plausible biosynthetic pathway of the eight different skeletons was shown in Scheme 2. It was proposed that 7-hydroxy-13-dehydroxypaxilline was the main precursor of all the paxilline-type indole-terpenoids, which could undergo prenylation, dehydration, methoxylation, and cyclization occurred to afford the other analogs.

### Antidiabetic activity assay

**Figure 5:**
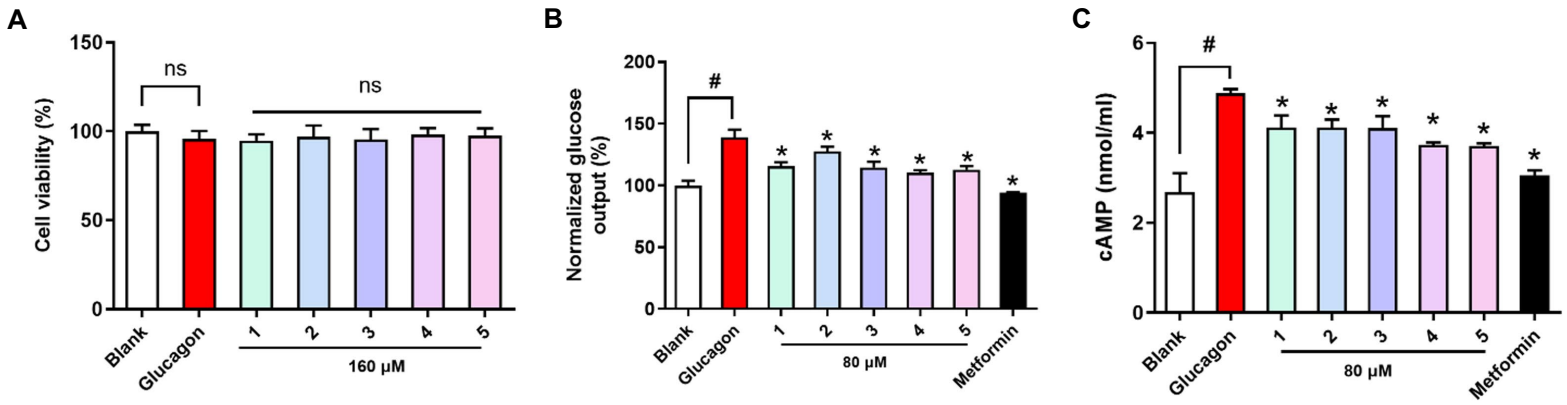
Viability and antidiabetic effects of compounds **1**-**5** against primary hepatocytes. **(A)** Cell viability **(B)** hepatic glucose output level; **(C)** cAMP contents in primary hepatocytes treated with glucagon (100 nM). Metformin (1 mM) as the positive control. ^ns^*p* > 0.05 vs. Blank, *p < 0.05 vs. Glucagon, ^#^*p* < 0.05 vs. Blank.

All the compounds were evaluated for cell viability at a concentration of 160 μM, and with this result, all the tested compounds (cell viability >90%) were selected for subsequent glucose output inhibition experiment (Figure 5). Glucose output in response to all the nontoxic compounds was measured to assess the antidiabetic effects in hepatocytes. Glucagon promotes hepatic glycogenolysis and increases hepatic gluconeogenesis, and we showed that glucagon challenge increased hepatic glucose output. Compounds **1-5** inhibited hepatic glucose output and their EC_50_ values (67.23, 102.1, 49.46, 25.20, and 35.96 μM) were higher than that of the positive control metformin (EC_50_ = 5.09 μM). Cyclic AMP (cAMP) as an intracellular second messenger is crucial for glucagon-induced hepatic glucose production. Glucagon challenge increased intracellular cAMP content, while compounds **1**-**5** treatment suppressed cAMP accumulation in hepatocytes. The results suggested that tested compounds inhibited hepatic glucose output may be by suppression hepatic cAMP accumulation.

## Conclusion

In our previous study, nine new indole-diterpenoids were isolated from the secondary metabolites of *Penicillium* sp. HFF16 and evaluated their anti-inflammatory and hypoglycemic activities. The results showed that encindolene C had the best anti-inflammatory activity compared with other compounds in RAW.2647 cells stimulated by LPS. Through simple structural analysis, it was speculated that the existence of prenyl group was beneficial to the improvement of anti-inflammatory activity. In HepG2 cells stimulated by glucagon, encindolene L showed the best inhibitory activity on hepatic glycogen export compared with other compounds. Structural analysis showed that encindolene L also had a prenyl group and indole and diterpene did not form a fused ring structure, again suggesting the importance of prenyl group in improving the biological activity of compounds. In consideration of such valuable work and the fact that the compound belongs to the tryptophan pathway of biosynthesis, the strain is subjected to secondary fermentation after a small amount of tryptophan is added to a rice culture medium. Four new indole-diterpenoid encindolenes I-L containing prenyl moieties were isolated and identified. Hypoglycemic activity was evaluated by mouse primary hepatocytes, and the results showed that encindolenes I-L could inhibit the increase of cAMP production induced by glucagon and reduce hepatic glucose output, thus exerting hypoglycemic effect. From the structural analysis, it was found that the compounds containing semi-acetal group had the worst hypoglycemic activity and the dehydration compound had the best activity, suggesting that semi-acetal group was harmful to biological activity. The comparison of the structure and activity results of encindolenes I and J suggests that the different substitution positions of the prenyl group have a significant effect on the activity.

## Data availability statement

The original contributions presented in the study are included in the article/Supplementary material, further inquiries can be directed to the corresponding author.

## Ethics statement

The animal study was reviewed and approved by All experiments and animal care conducted in accordance with the Provision and General Recommendation of Chinese Experimental Animals Administration Legislation and were approved by the Animal Ethics Committee of Shandong Agriculture University.

## Author contributions

NX and LZ contributed to bioactivity assay and revised the manuscript. GP conceived and designed the experiments and was involved in isolation of compounds. XjL, XyL, MZ, FL, and XlL contributed to isolation and collection of the NMR data of compounds. XK, JB, XyL, DK, and GH performed strain fermentation and extraction. FK supervised the study and prepared the manuscript. All authors contributed to the article and approved the submitted version.

## Funding

This study was financially supported by the Natural Science Foundation of Shandong Province (ZR2021MB087), the National Natural Science Foundation of China (82004014), the Shandong traditional Chinese Medicine Science and Technology Project (2021Q083), the Innovation and entrepreneurship training program for college students in Shandong Province (202210439005, 202214039008), the Specific research project of Guangxi for research bases and talents (AD18126005), and the Natural Science Foundation of Guangxi (2021GXNSFBA075036).

## Conflict of interest

The authors declare that the research was conducted in the absence of any commercial or financial relationships that could be construed as a potential conflict of interest.

## Publisher’s note

All claims expressed in this article are solely those of the authors and do not necessarily represent those of their affiliated organizations, or those of the publisher, the editors and the reviewers. Any product that may be evaluated in this article, or claim that may be made by its manufacturer, is not guaranteed or endorsed by the publisher.
