# Supplementary material for: Prenylated indole-terpenoids with antidiabetic activities from Penicillium sp. HFF16 from the rhizosphere soil of Cynanchum bungei Decne
Source: Front Microbiol. 2023 Mar 2;14:1099103. doi: 10.3389/fmicb.2023.1099103 (PMC10018213; doi:10.3389/fmicb.2023.1099103)
Supplement: Supplementary file 1 [file Data_Sheet_1.PDF]

Supplementary Material

Prenylated Indole-Terpenoids With Antidiabetic Activities From *Penicillium* sp. HFF16 From the Rhizosphere Soil of *Cynanchum bungei* Decne.

Xijin Liu,[1#] Fandong Kong,[2#] Na Xiao,[3#] Xiaoyu Li,[1] Mingyu Zhang,[1] Fujin Lv,[1] Xiaolin Liu,[1] Xiangchuan Kong,[1] Jing Bi,[1] Xinyi Lu,[1] Daqing Kong,[1] Gangping Hao,[1] Liman Zhou,[2] Guojun Pan [1*]

[1] *College of Life Sciences, Shandong First Medical University & Shandong Academy of Medical Sciences, Tai'an, Shandong 271000, China.*

[2] *Key Laboratory of Chemistry and Engineering of Forest Products, State Ethnic Affairs Commission, Guangxi Key Laboratory of Chemistry and Engineering of Forest Products, Guangxi Collaborative Innovation Center for Chemistry and Engineering of Forest Products, School of Chemistry and Chemical Engineering, Guangxi Minzu University, Nanning 530006, China.*

[3] *State Key Laboratory of Crop Biology, college of Agronomy, Shandong Agriculture University, Tai'an, Shandong 271018, China.*

List of Supporting Information

Figure S1. ^{1}H NMR (400 MHz) spectrum of compound **1** in MeOD S4

Figure S2. ^{13}C NMR (100 MHz) spectrum of compound **1** in MeOD S4

Figure S3. HSQC spectrum of compound **1** in MeOD........S5

Figure S4. ^{1}H-^{1}H COSY spectrum of compound **1** in MeOD S5

Figure S5. HMBC spectrum of compound **1** in MeOD S6

Figure S6. ROESY spectrum of compound **1** in MeOD S6

Figure S7. HRESIMS of compound **1**........S7

Figure S8. ^{1}H NMR (400 MHz) spectrum of compound **2** in MeOD S8

Figure S9. ^{13}C NMR (100 MHz) spectrum of compound **2** in MeOD S8

Figure S10. HSQC spectrum of compound **2** in MeOD S9

Figure S11. ^{1}H-^{1}H COSY spectrum of compound **2** in MeOD S9

Figure S12. HMBC spectrum of compound **2** in MeOD S10

Figure S13. ROESY spectrum of compound **2** in MeOD S10

Figure S14. HRESIMS of compound **2**........S11

Figure S15. ^{1}H NMR (400 MHz) spectrum of compound **3** in MeOD S12

Figure S16. ^{13}C NMR (100 MHz) spectrum of compound **3** in MeOD S12

Figure S17. HSQC spectrum of compound **3** in MeOD........S13

Figure S18. ^{1}H-^{1}H COSY spectrum of compound **3** in MeOD S13

Figure S19. HMBC spectrum of compound **3** in MeOD S14

Figure S20. ROESY spectrum of compound **3** in MeOD S14

Figure S21. HRESIMS of compound **3**........S15

Figure S22. ^{1}H NMR (400 MHz) spectrum of compound **4** in MeOD S16

Figure S23. ^{13}C NMR (100 MHz) spectrum of compound **4** in MeOD S16

Figure S24. HSQC spectrum of compound **4** in MeOD........S17

Figure S25. ^{1}H-^{1}H COSY spectrum of compound **4** in MeOD S17

Figure S26. HMBC spectrum of compound **4** in MeOD S18

Figure S27. ROESY spectrum of compound **4** in MeOD S18

Figure S28. HRESIMS of compound **4**........S19

Figure S29. HPLC conversion results of paspalitrem C to **2** and **3** in 0.1% trifluoroacetic acid

in methanol..S20

Figure S30. HPLC conversion results of compound **5** to **1** and **4** in 0.1% trifluoroacetic acid in methanol..S20

Figure S31. FT-IR spectrum of compound **1**..S21

Figure S32. FT-IR spectrum of compound **2**..S21

Figure S33. FT-IR spectrum of compound **3**..S22

Figure S34. FT-IR spectrum of compound **4**..S22

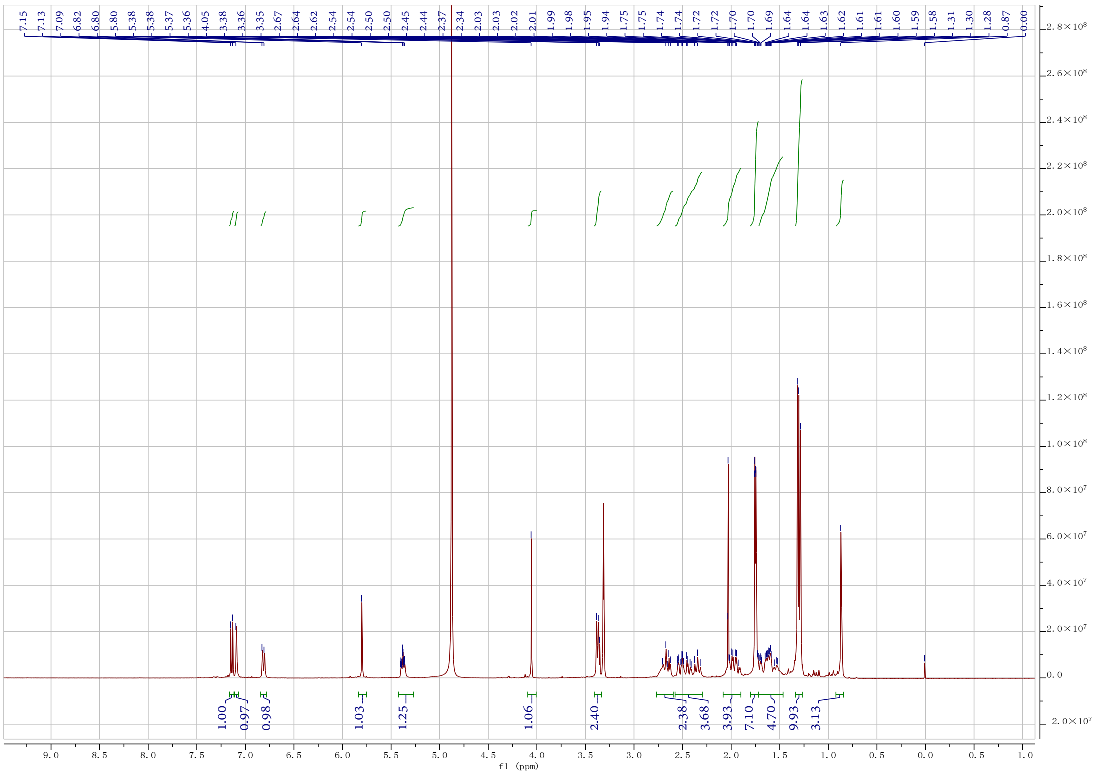

Figure S1. ^{1}H NMR (400 MHz) spectrum of compound **1** in MeOD

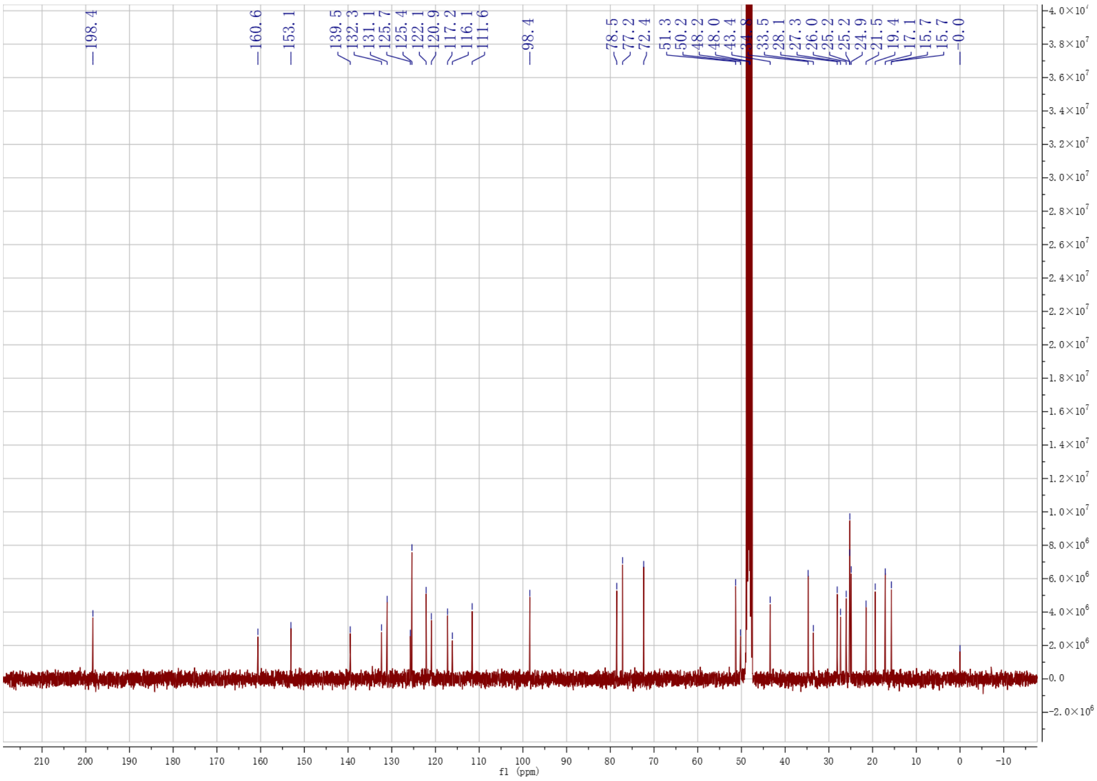

Figure S2. ^{13}C NMR (100 MHz) spectrum of compound **1** in MeOD

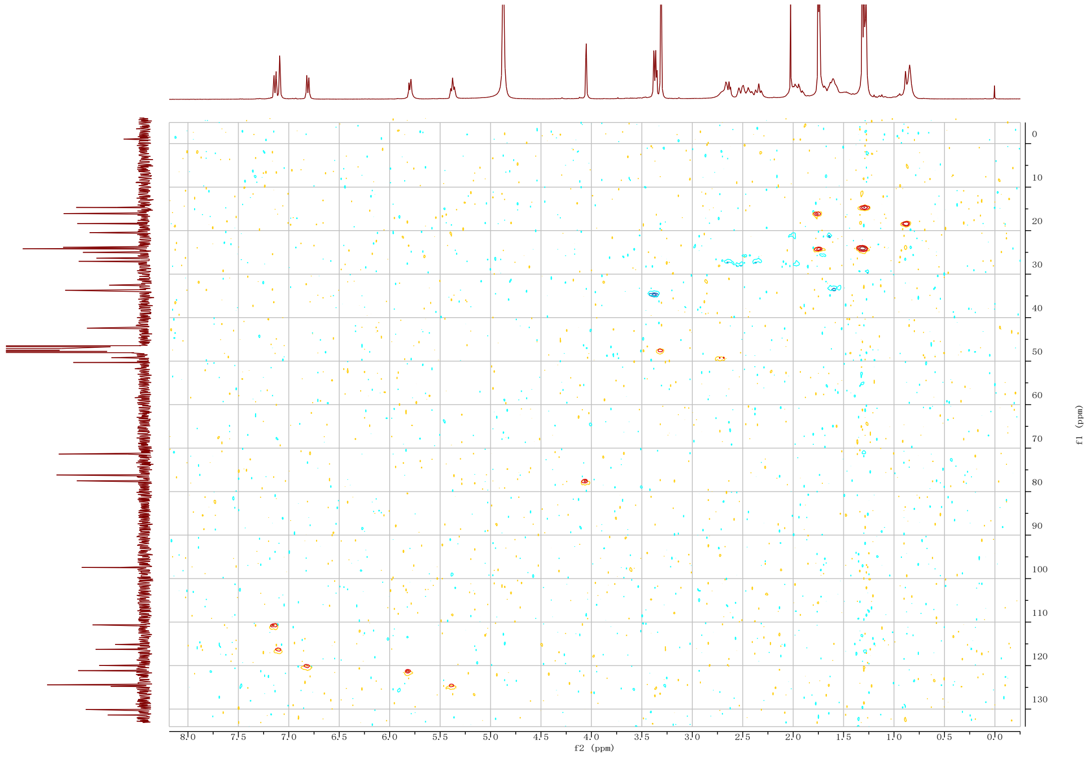

Figure S3. HSQC spectrum of compound **1** in MeOD

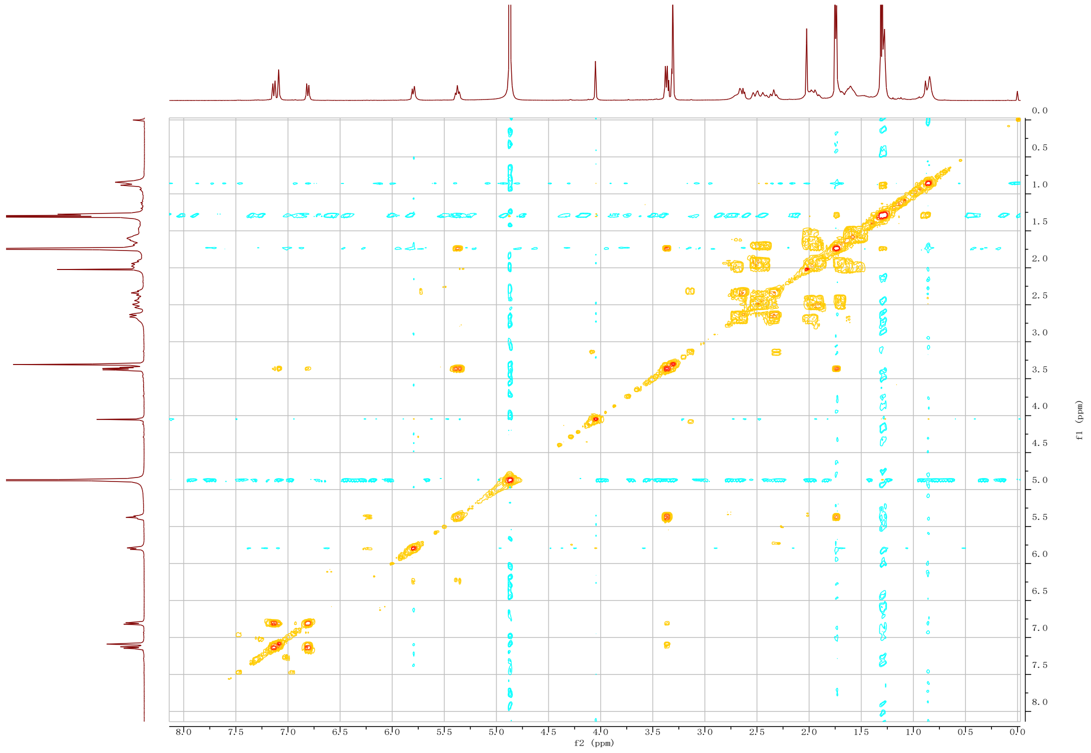

Figure S4. ^{1}H-^{1}H COSY spectrum of compound **1** in MeOD

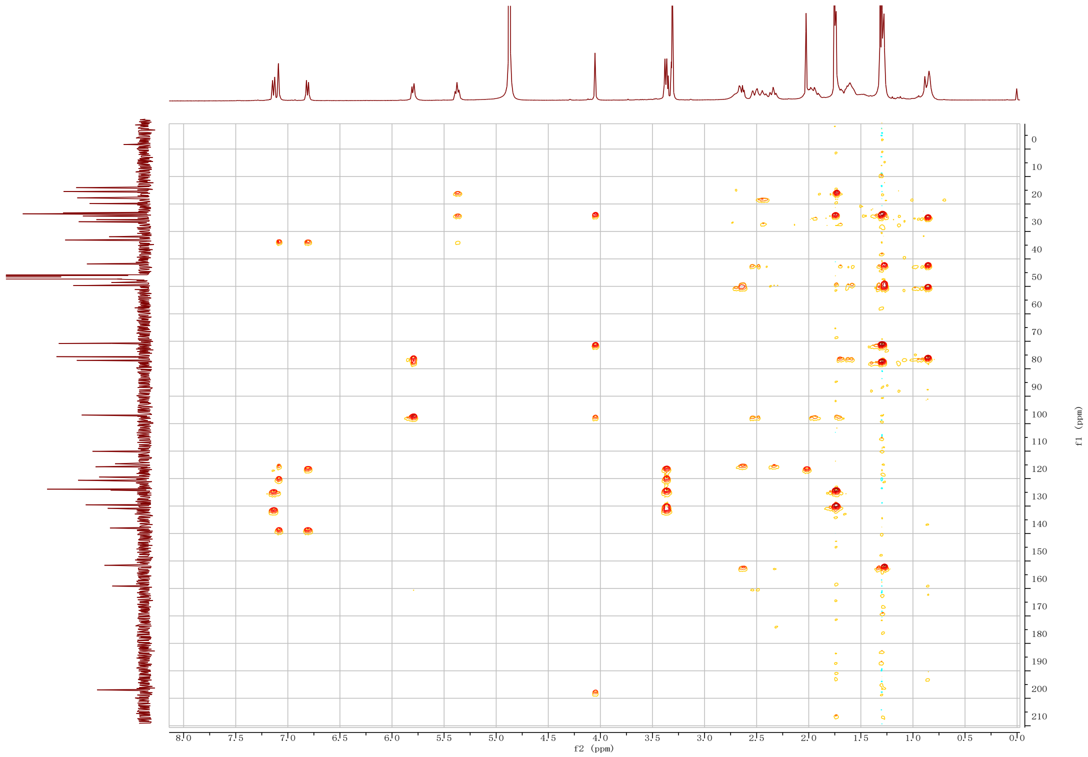

Figure S5. HMBC spectrum of compound **1** in MeOD

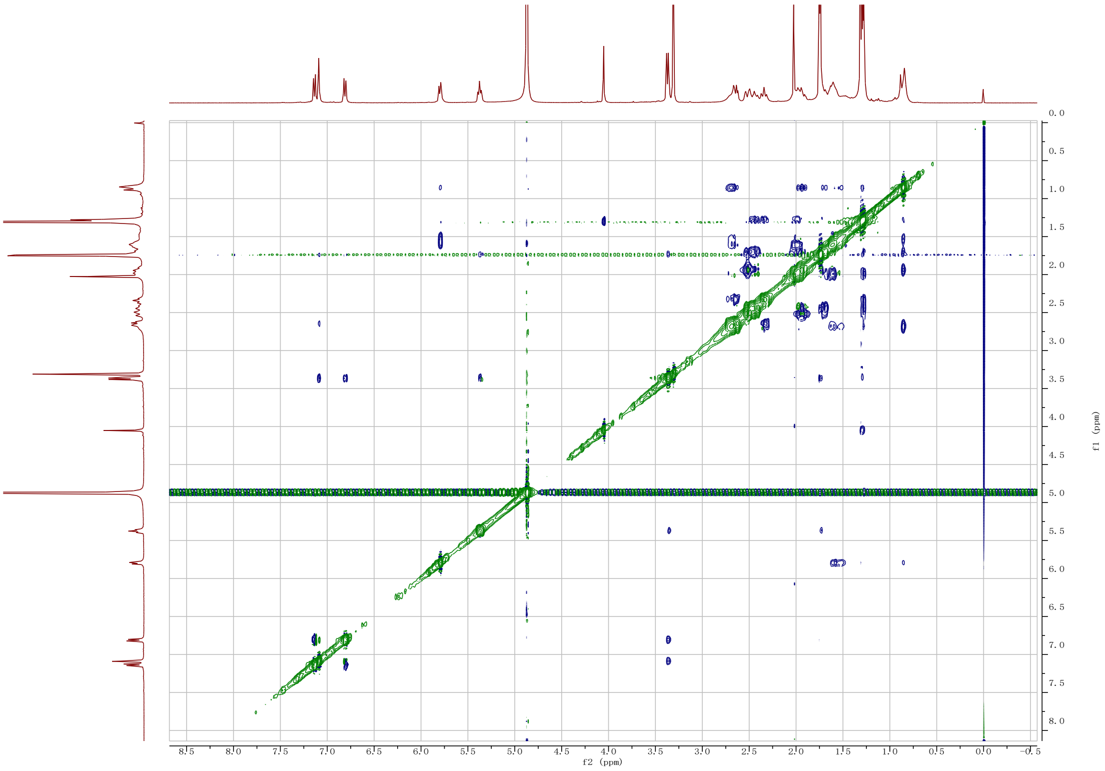

Figure S6. NOESY spectrum of compound **1** in MeOD

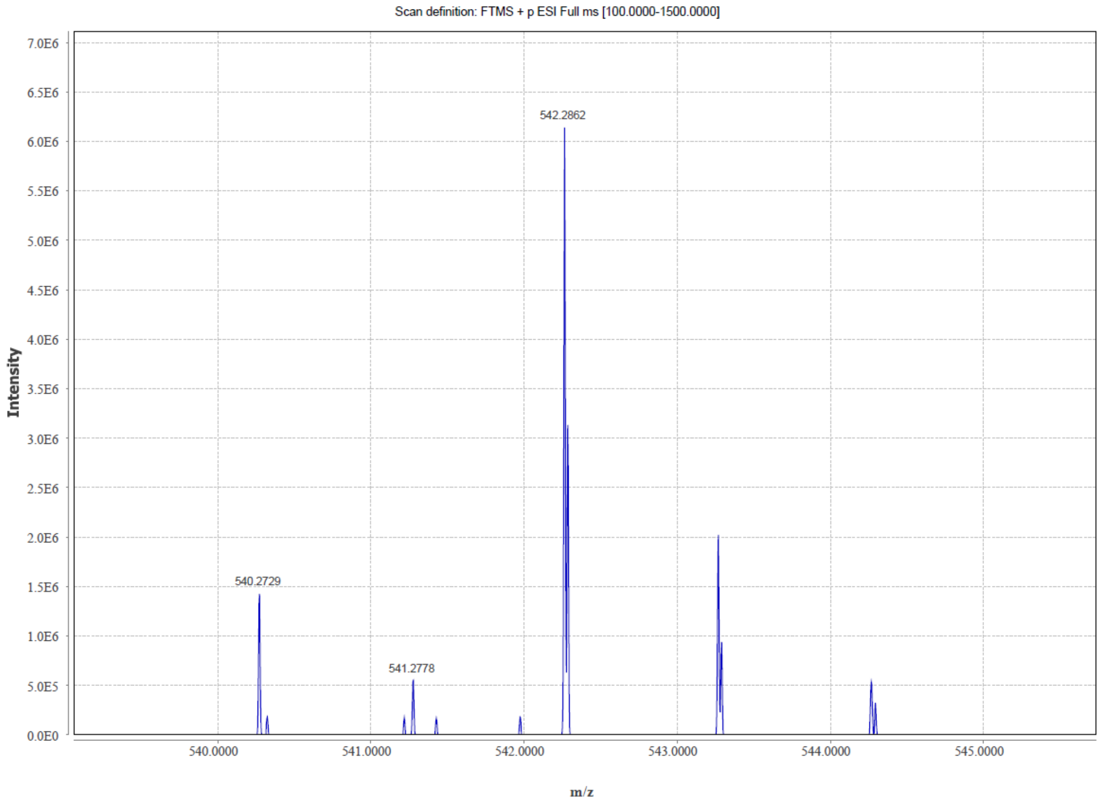

Figure S7. HRESIMS of compound **1**

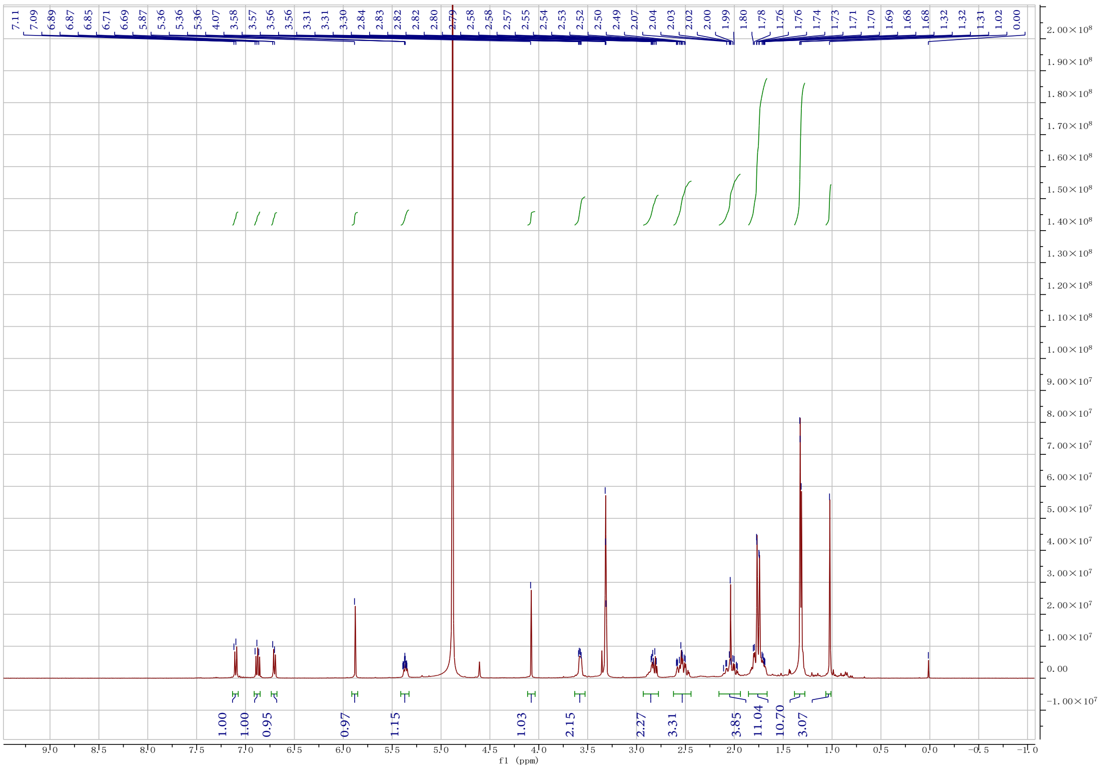

Figure S8. ^{1}H NMR (400 MHz) spectrum of compound **2** in MeOD

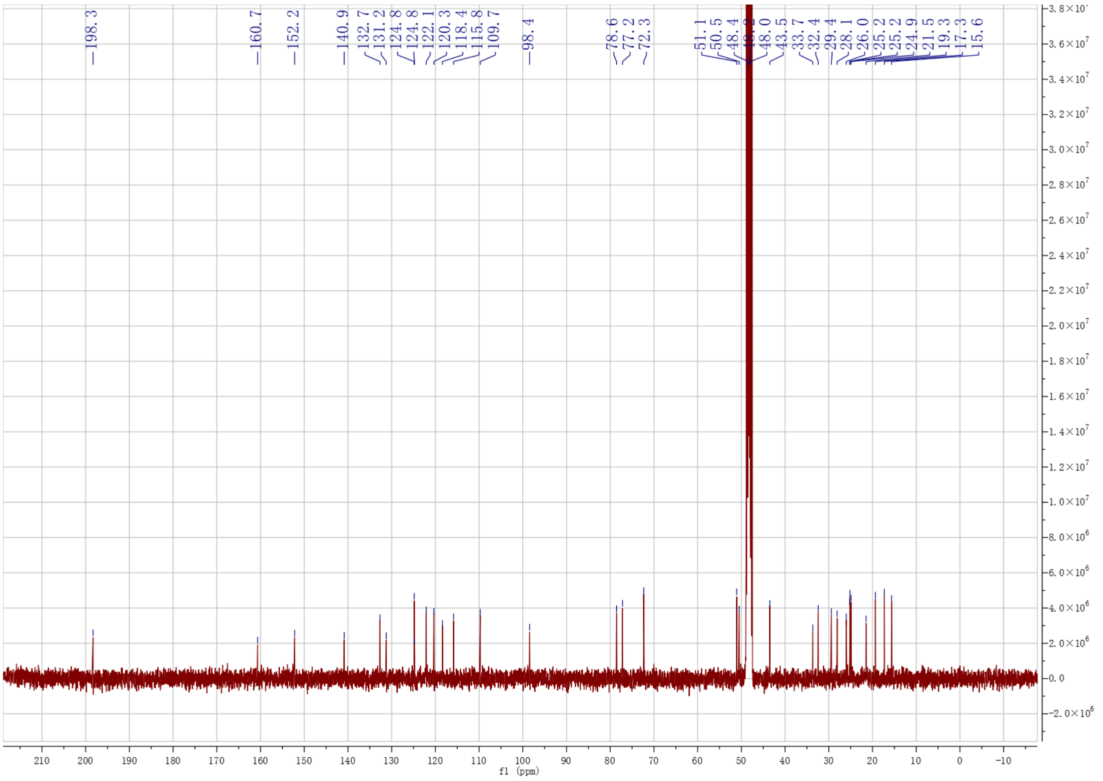

Figure S9. ^{13}C NMR (100 MHz) spectrum of compound **2** in MeOD

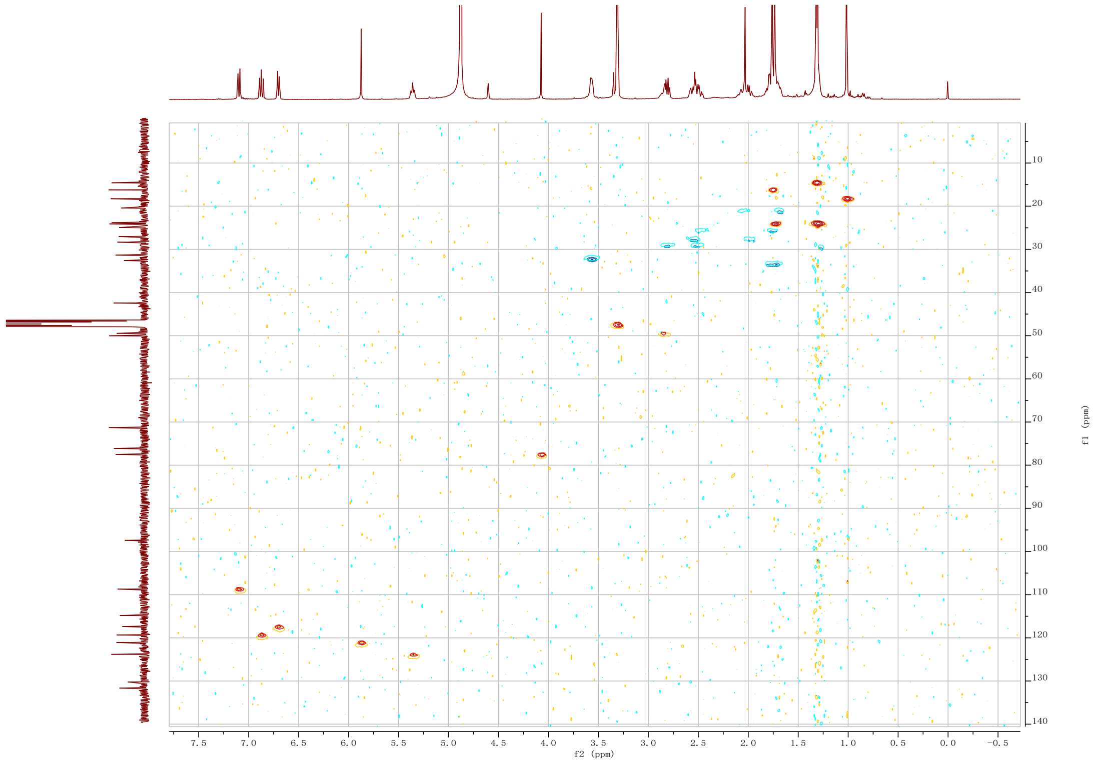

Figure S10. HSQC spectrum of compound **2** in MeOD

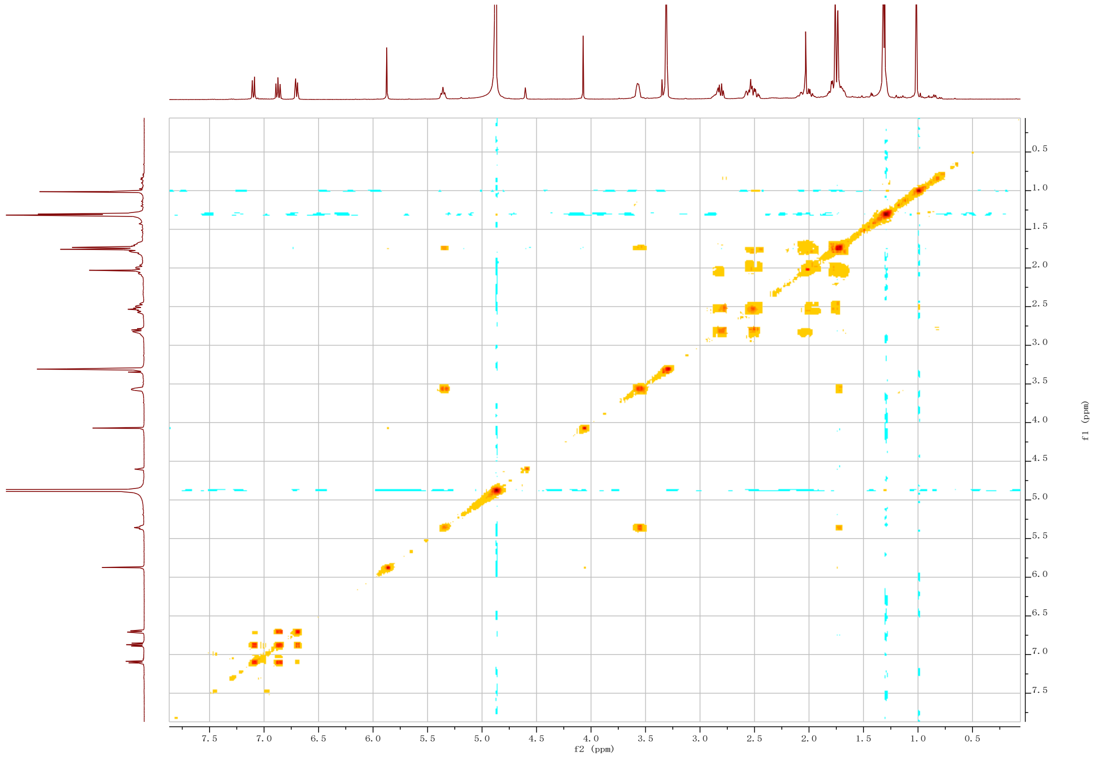

Figure S11. ^1H-^1H COSY spectrum of compound **2** in MeOD

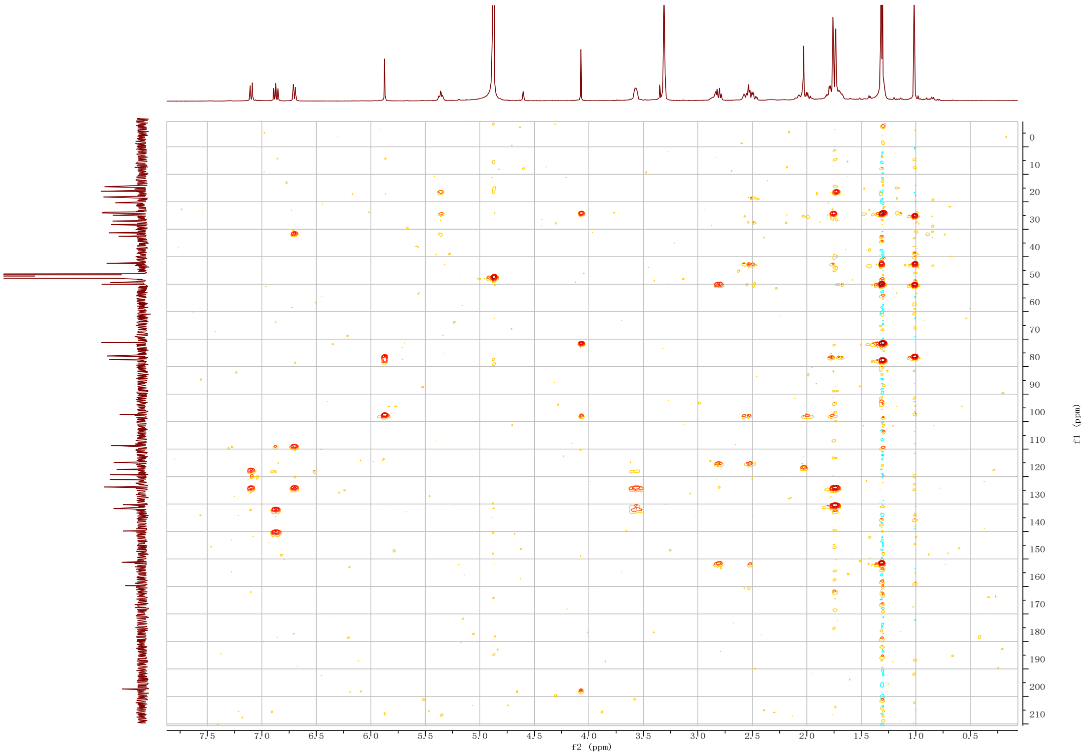

Figure S12. HMBC spectrum of compound **2** in MeOD

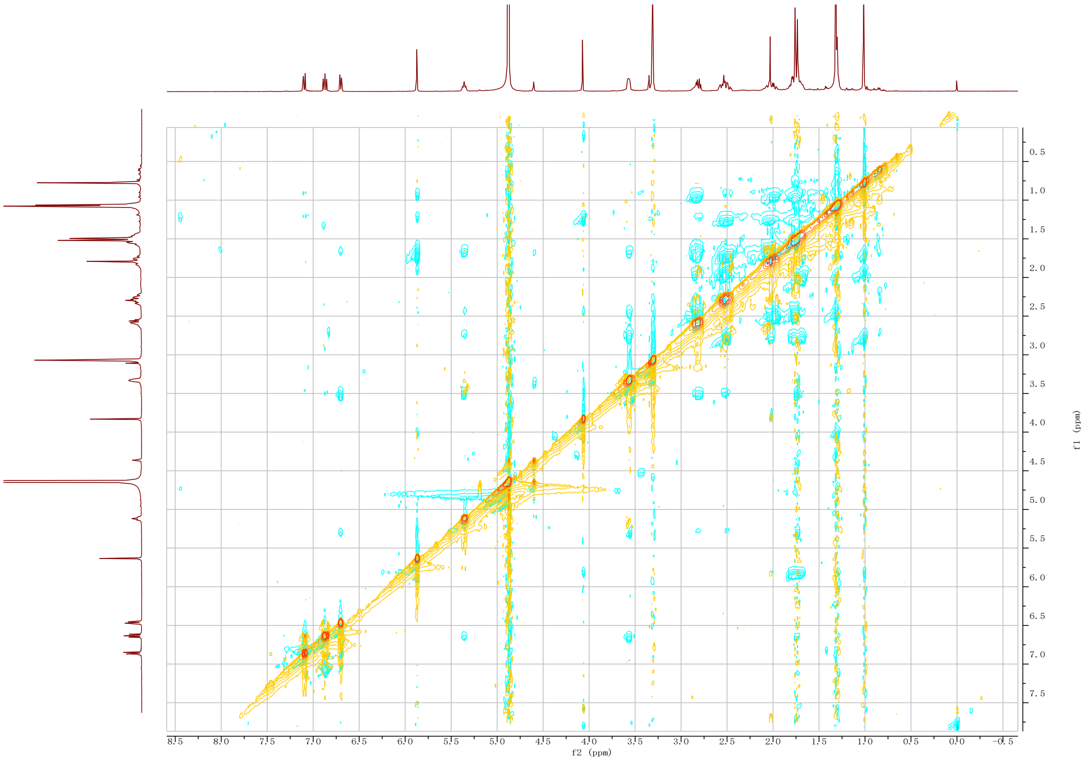

Figure S13. NOESY spectrum of compound **2** in MeOD

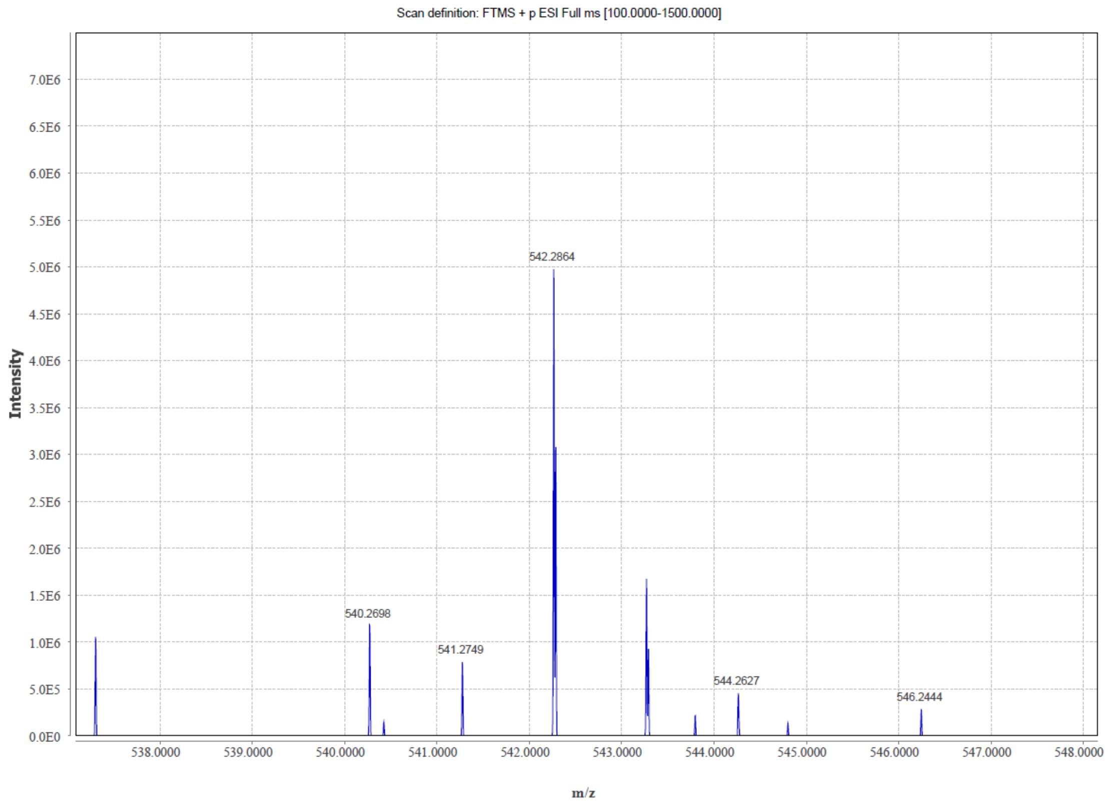

Figure S14. HRESIMS of compound **2**

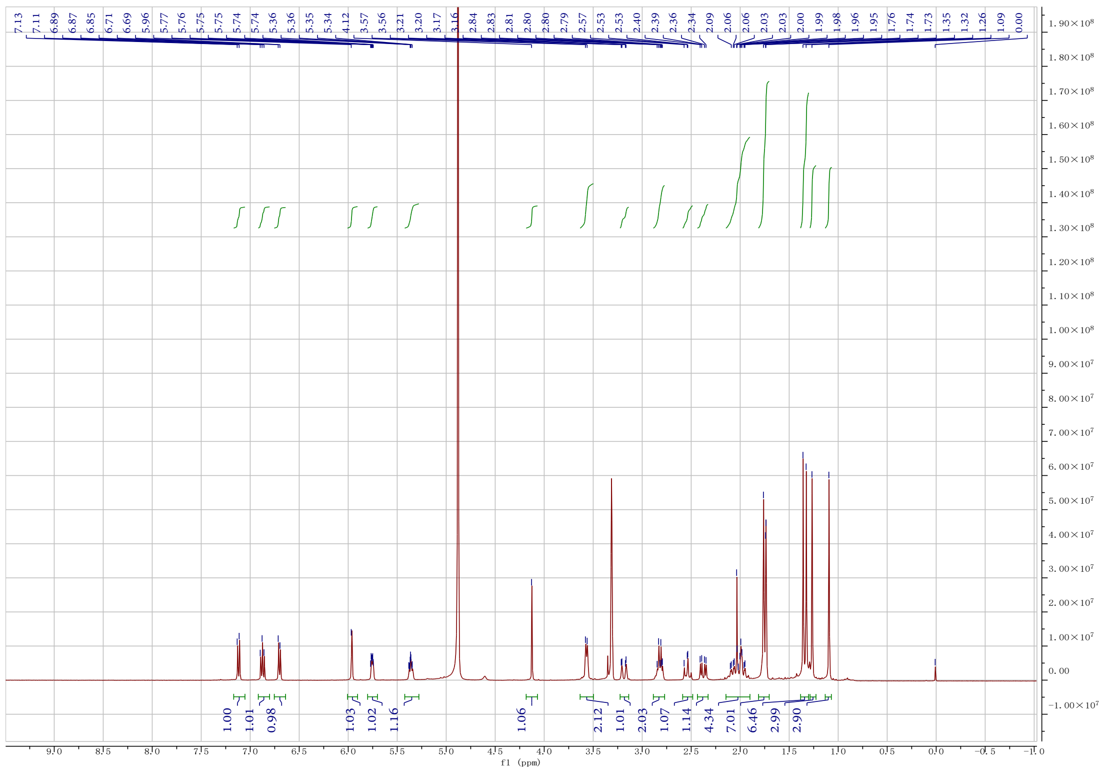

Figure S15. ^{1}H NMR (400 MHz) spectrum of compound **3** in MeOD

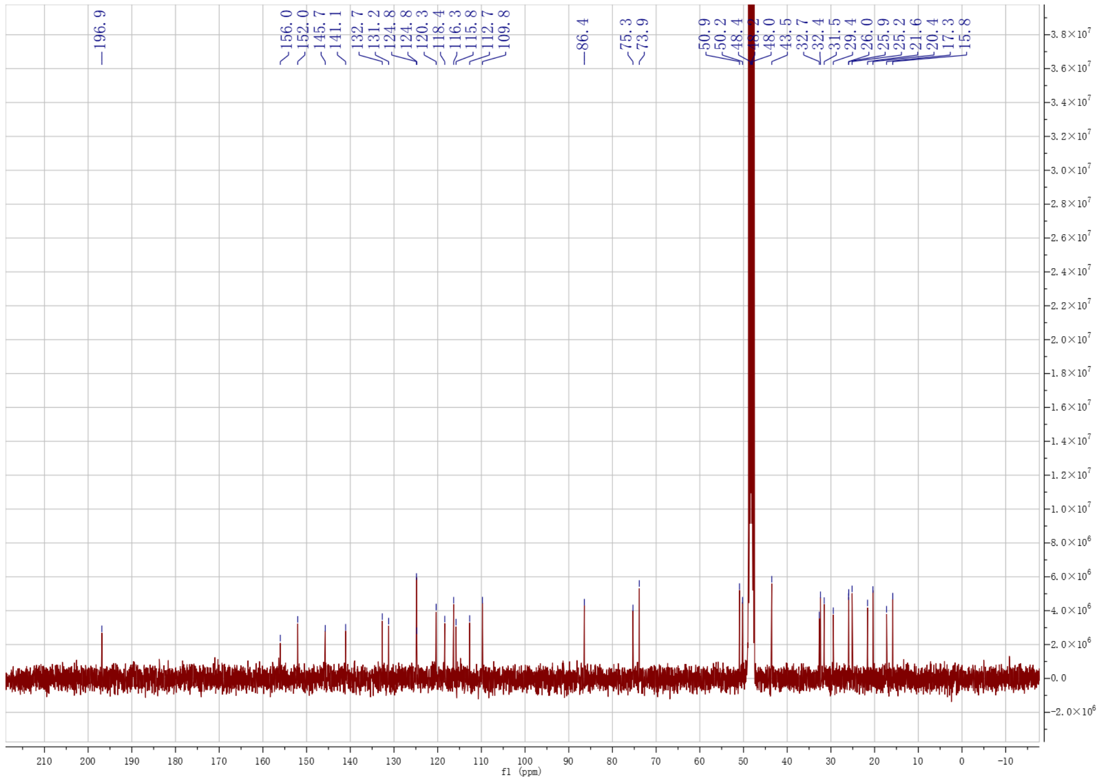

Figure S16. ^{13}C NMR (100 MHz) spectrum of compound **3** in MeOD

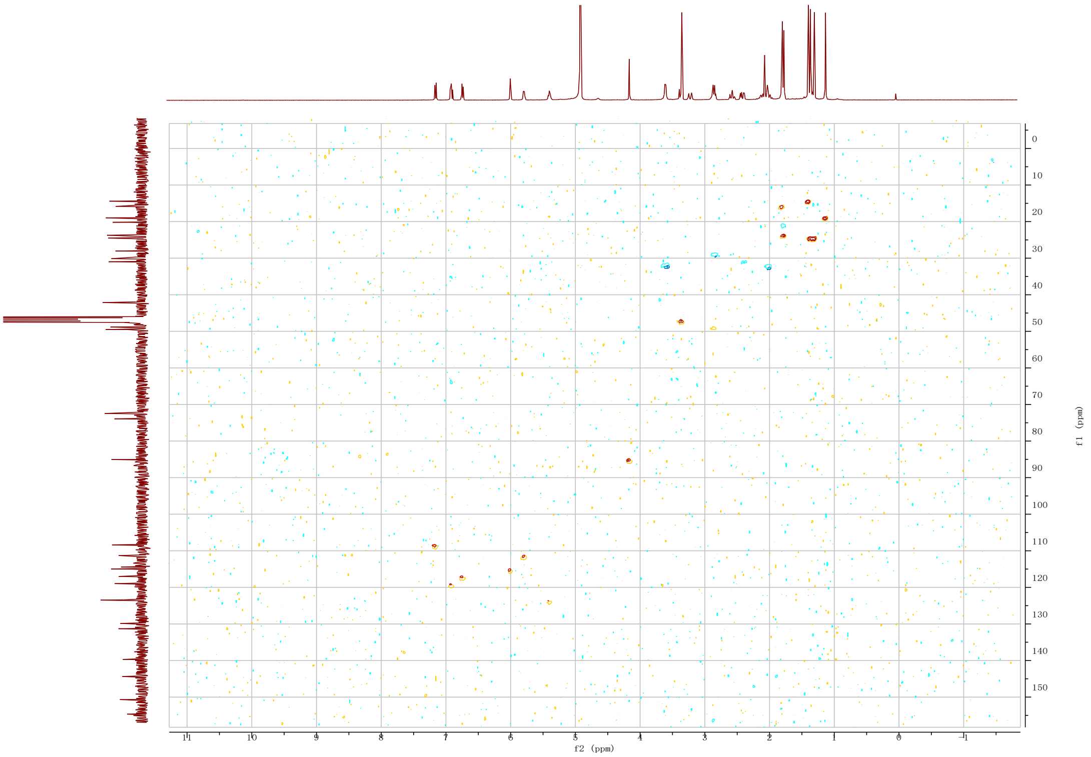

Figure S17. HSQC spectrum of compound **3** in MeOD

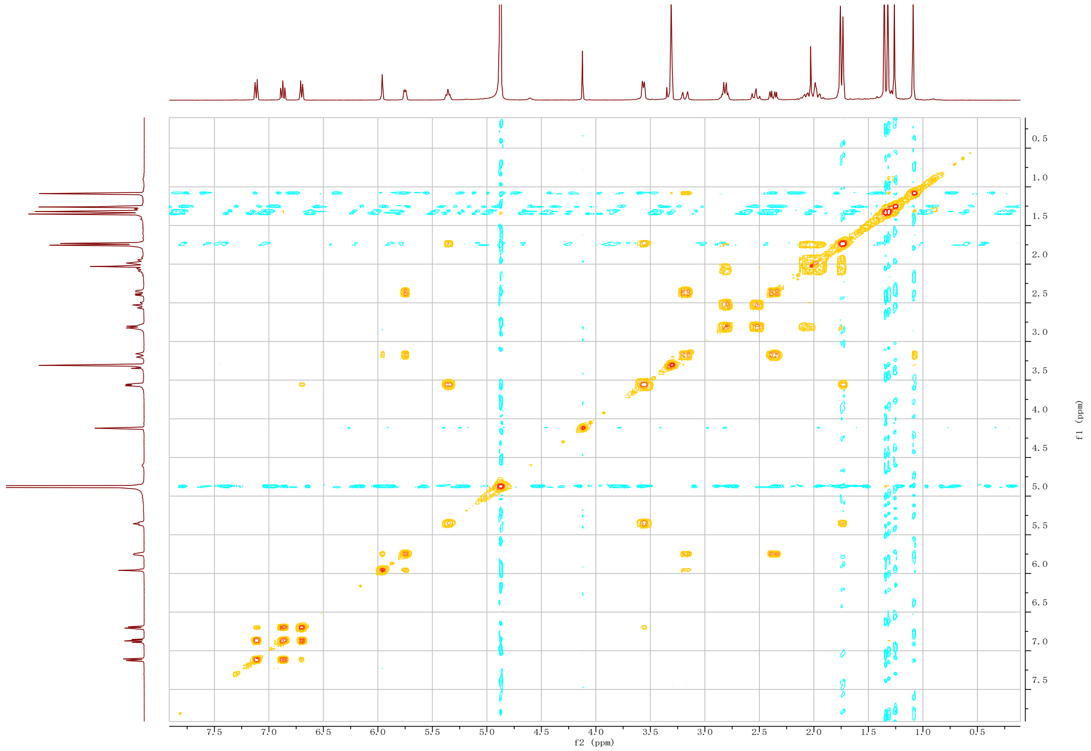

Figure S18. ^{1}H-^{1}H COSY spectrum of compound **3** in MeOD

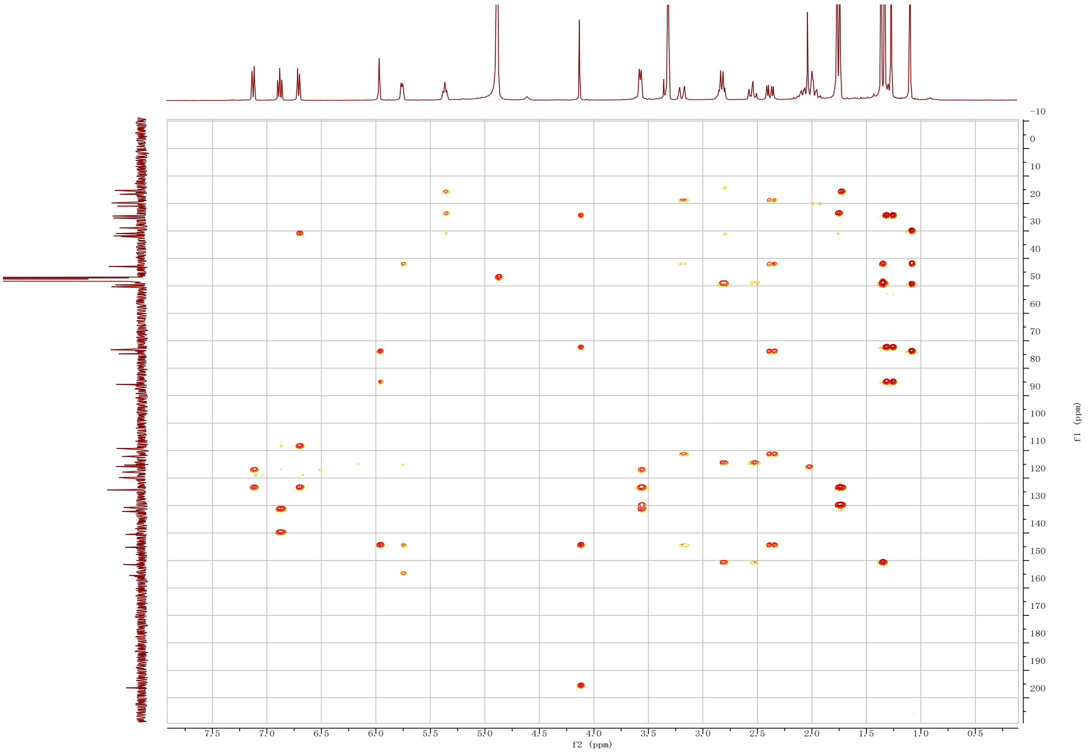

Figure S19. HMBC spectrum of compound **3** in MeOD

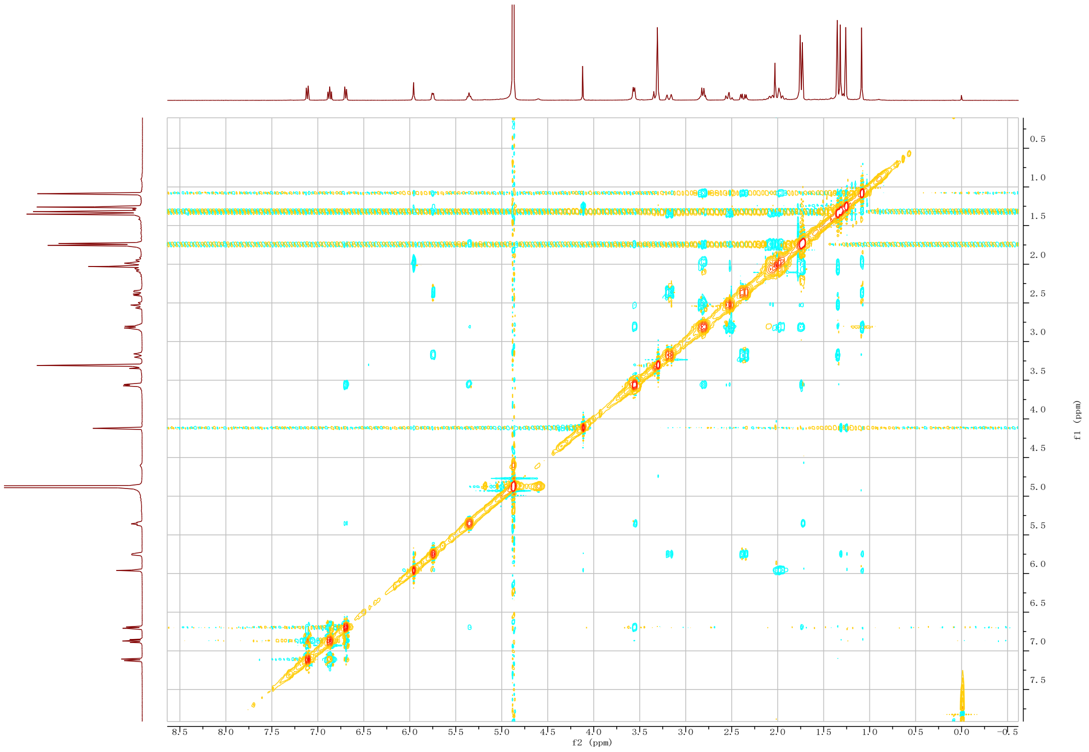

Figure S20. ROESY spectrum of compound **3** in MeOD

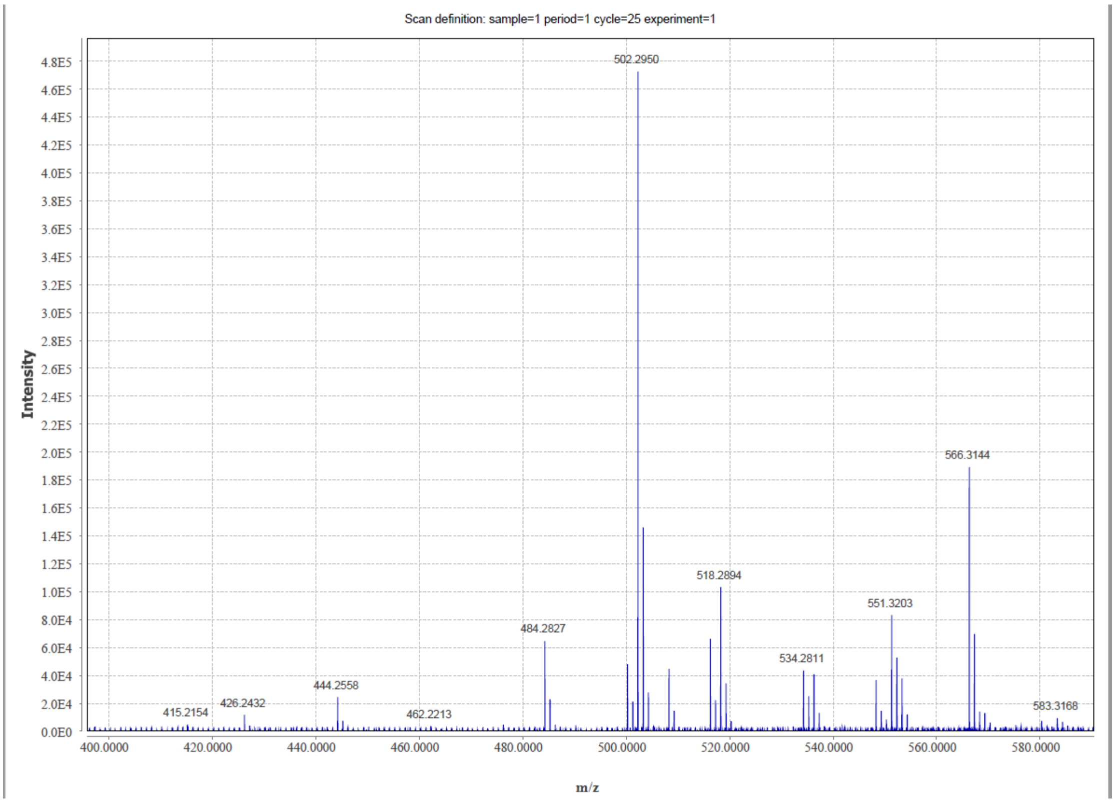

Figure S21. HRESIMS of compound **3**

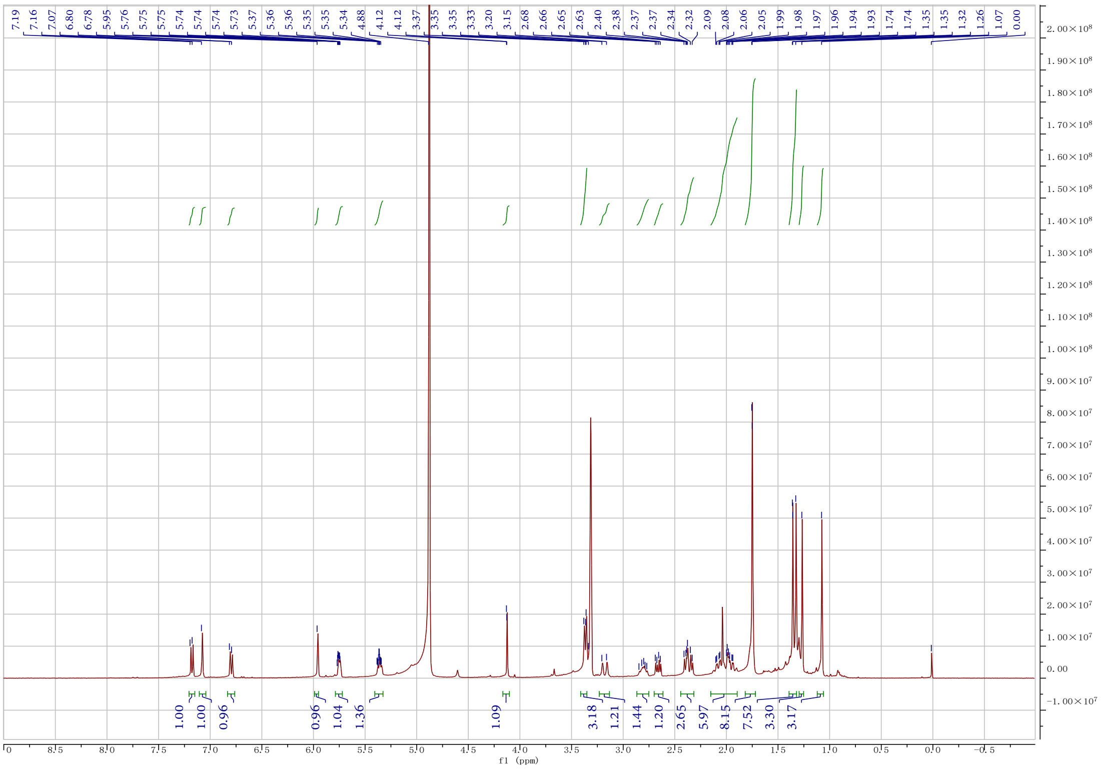

Figure S22. ^{1}H NMR (400 MHz) spectrum of compound **4** in MeOD

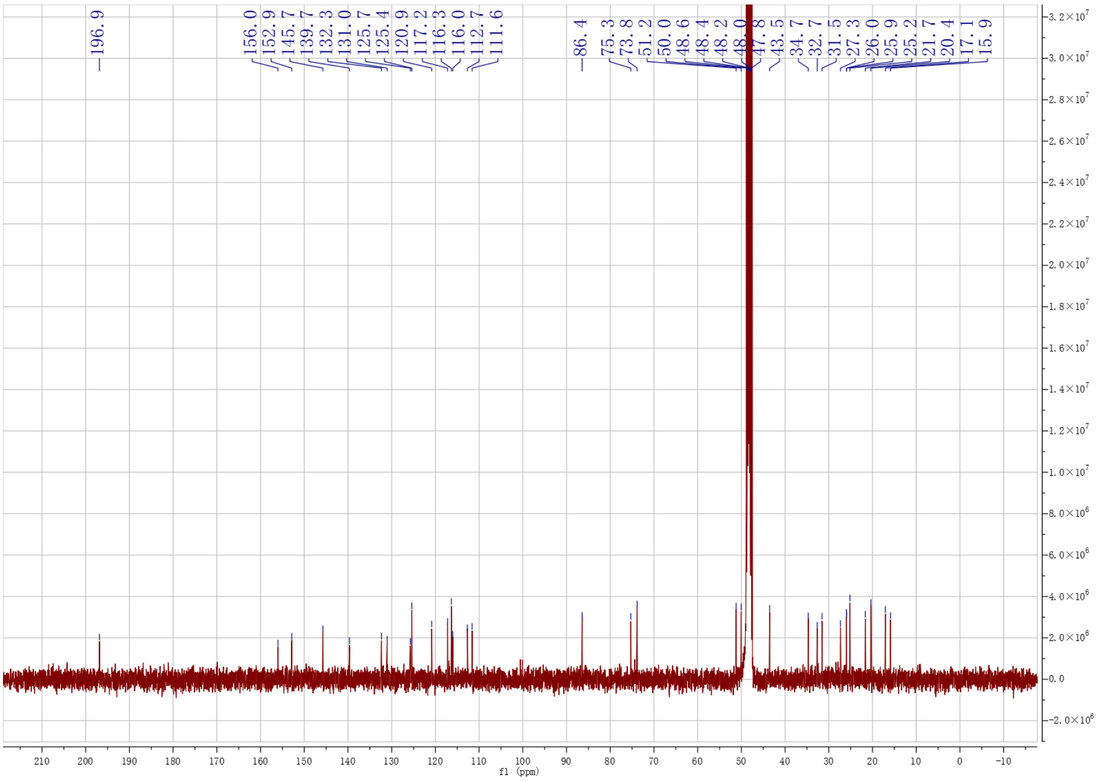

Figure S23. ^{13}C NMR (100 MHz) spectrum of compound **4** in MeOD

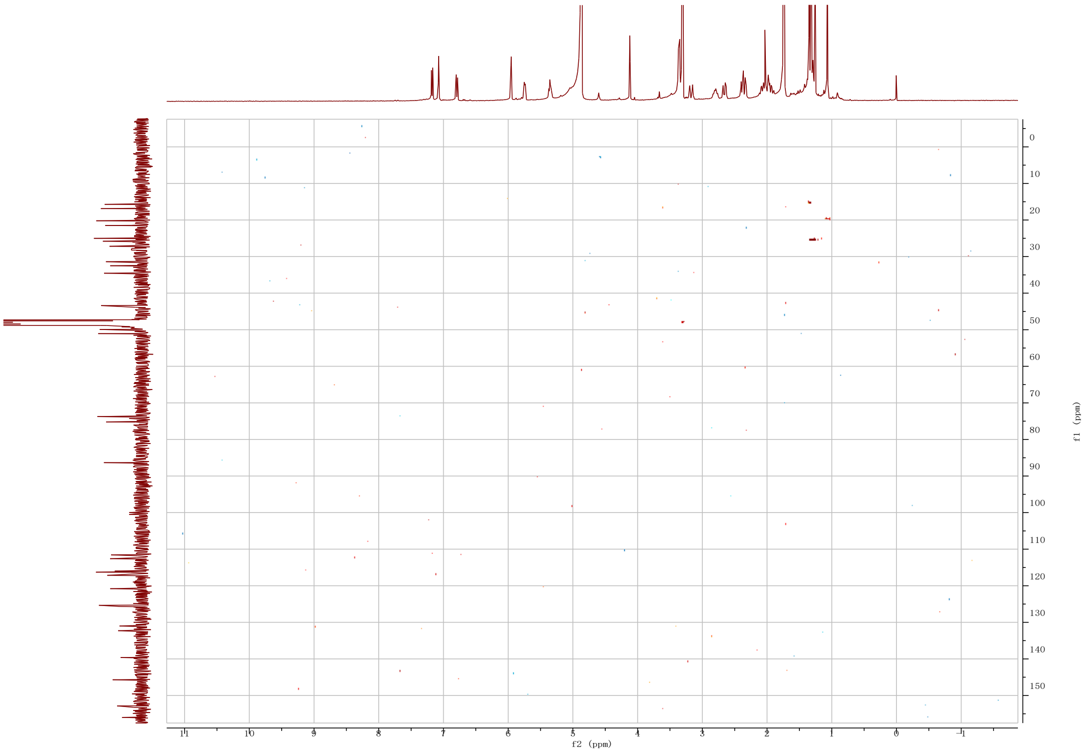

Figure S24. HSQC spectrum of compound **4** in MeOD

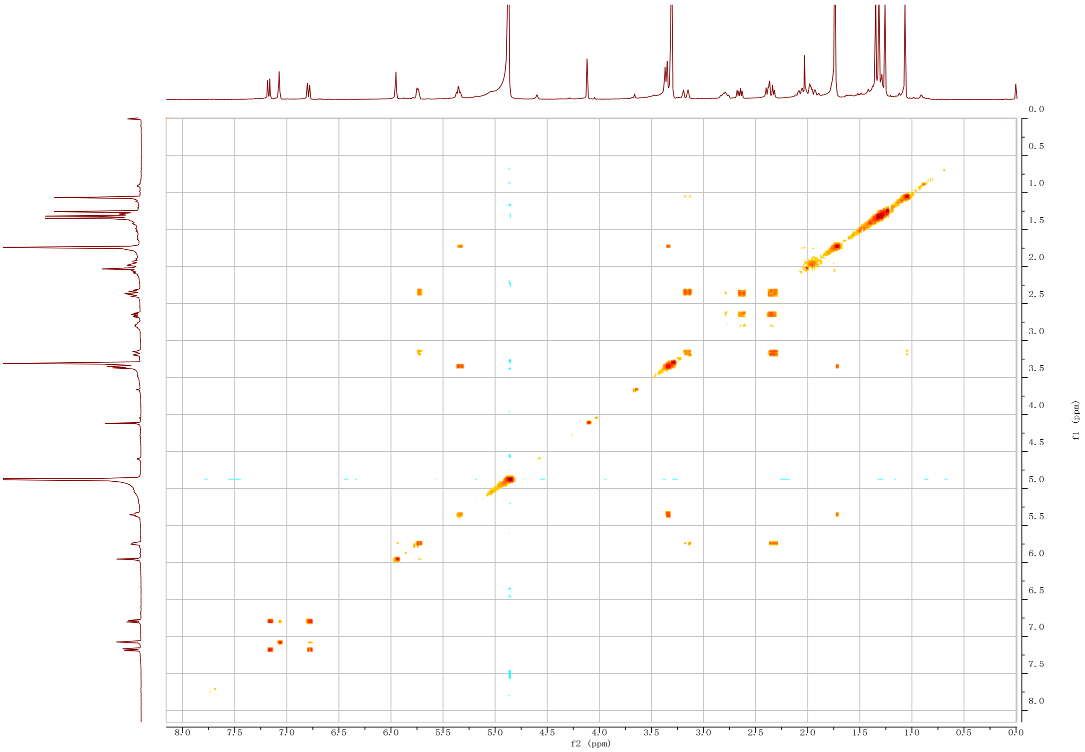

Figure S25. ^{1}H-^{1}H COSY spectrum of compound **4** in MeOD

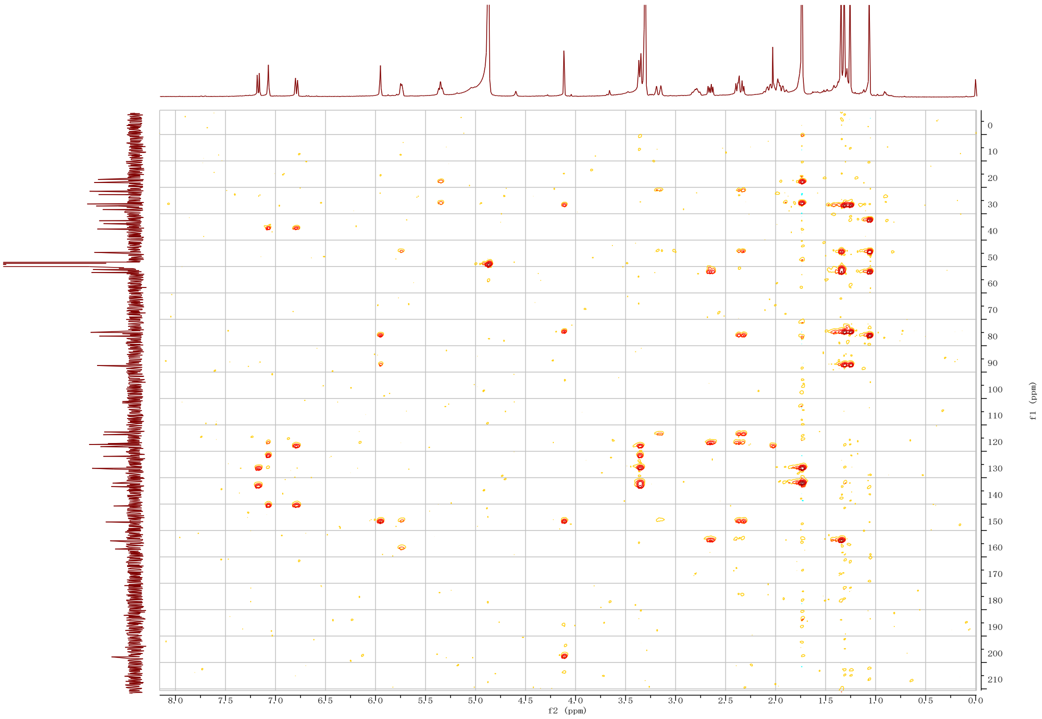

Figure S26. HMBC spectrum of compound **4** in MeOD

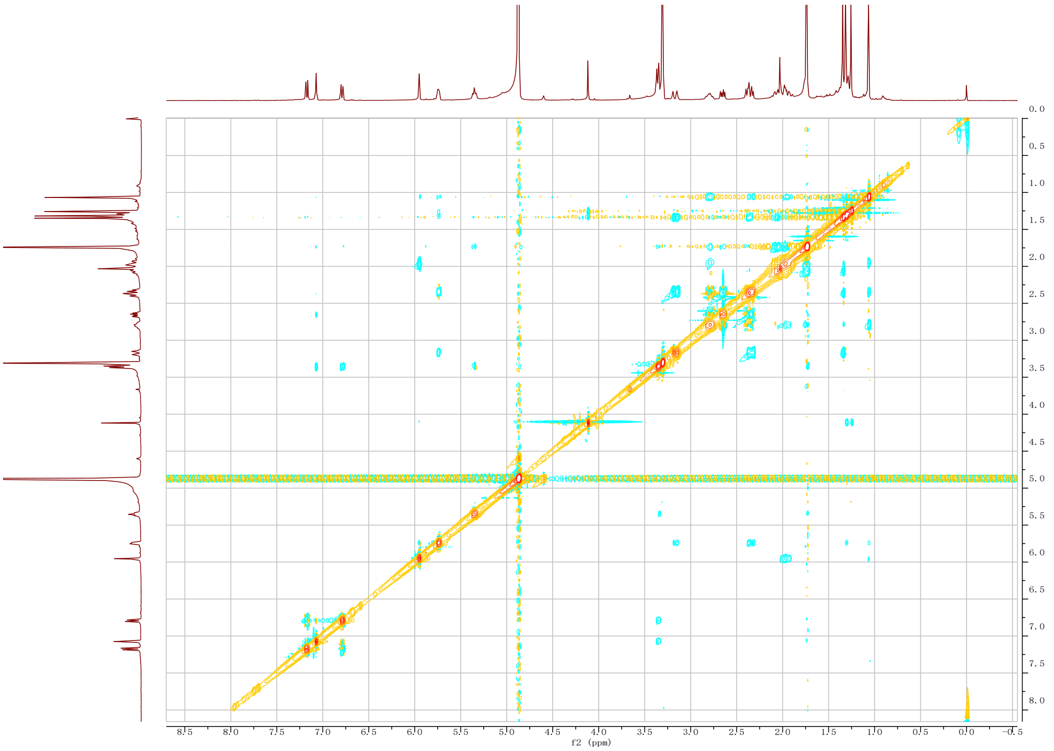

Figure S27. ROESY spectrum of compound **4** in MeOD

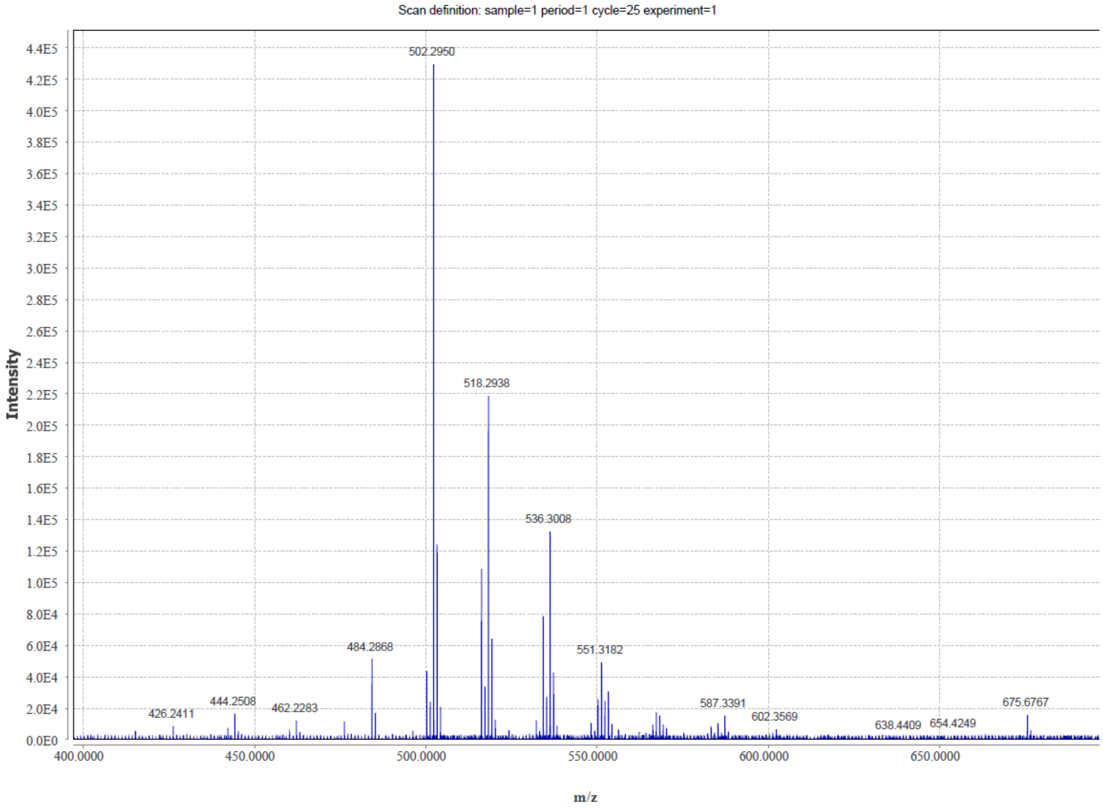

Figure S28. HRESIMS of compound **4**

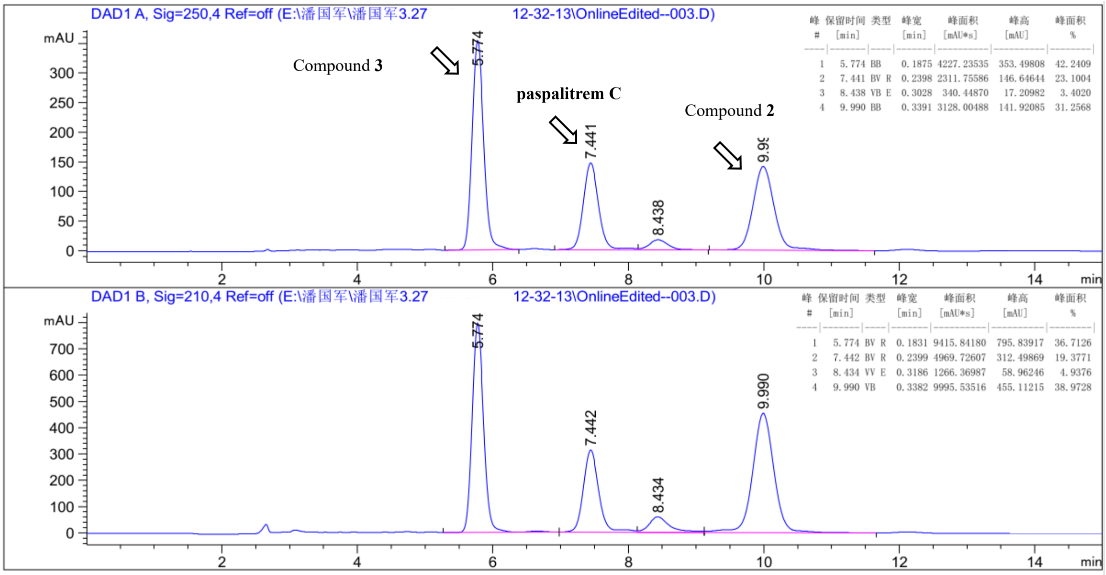

Figure S29. HPLC conversion results of paspalitrem C to **2** and **3** in 0.1% trifluoroacetic acid in methanol.

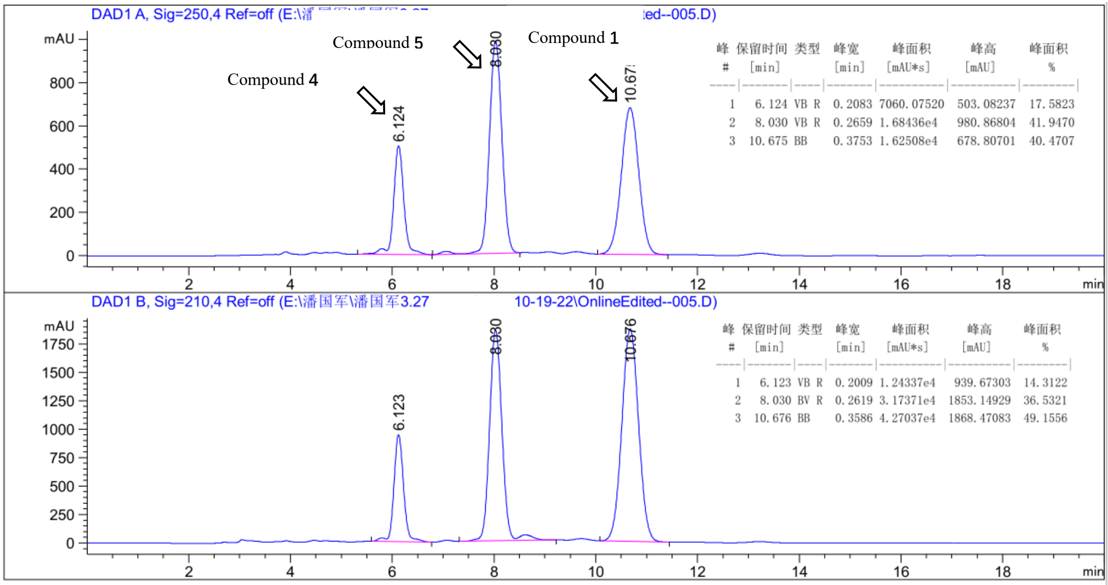

Figure S30. HPLC conversion results of compound **5** to **1** and **4** in 0.1% trifluoroacetic acid in methanol.

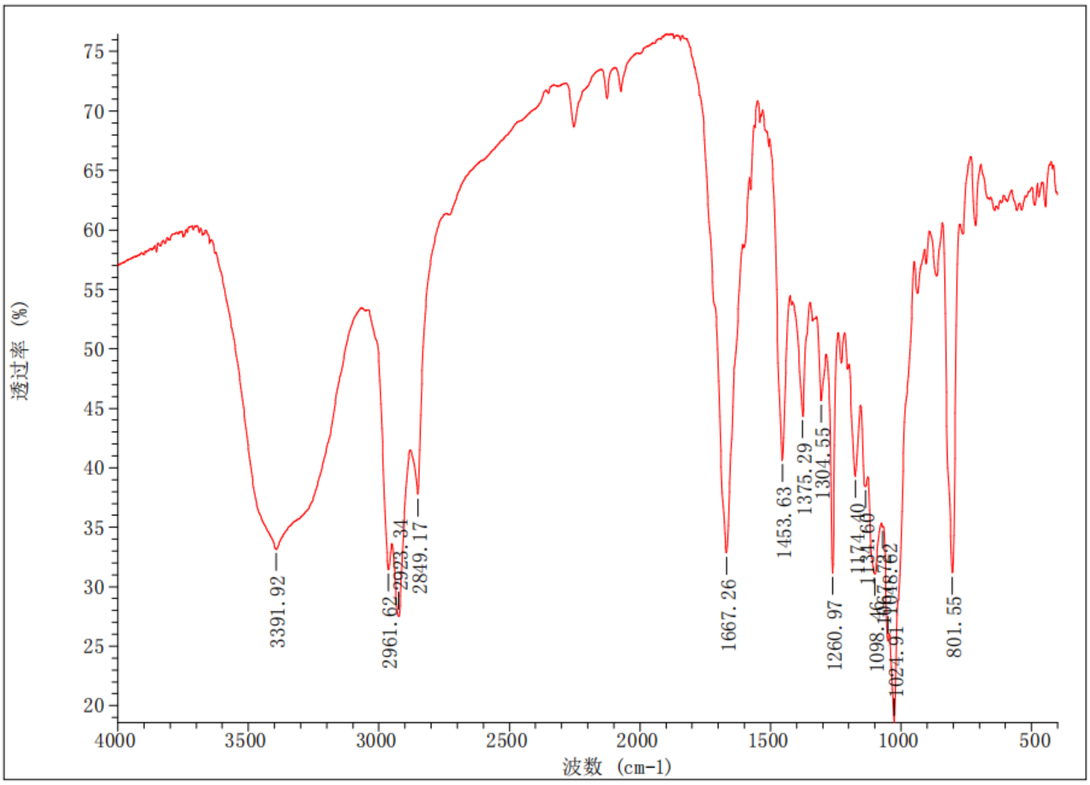

Figure S31. FT-IR spectrum of compound **1**.

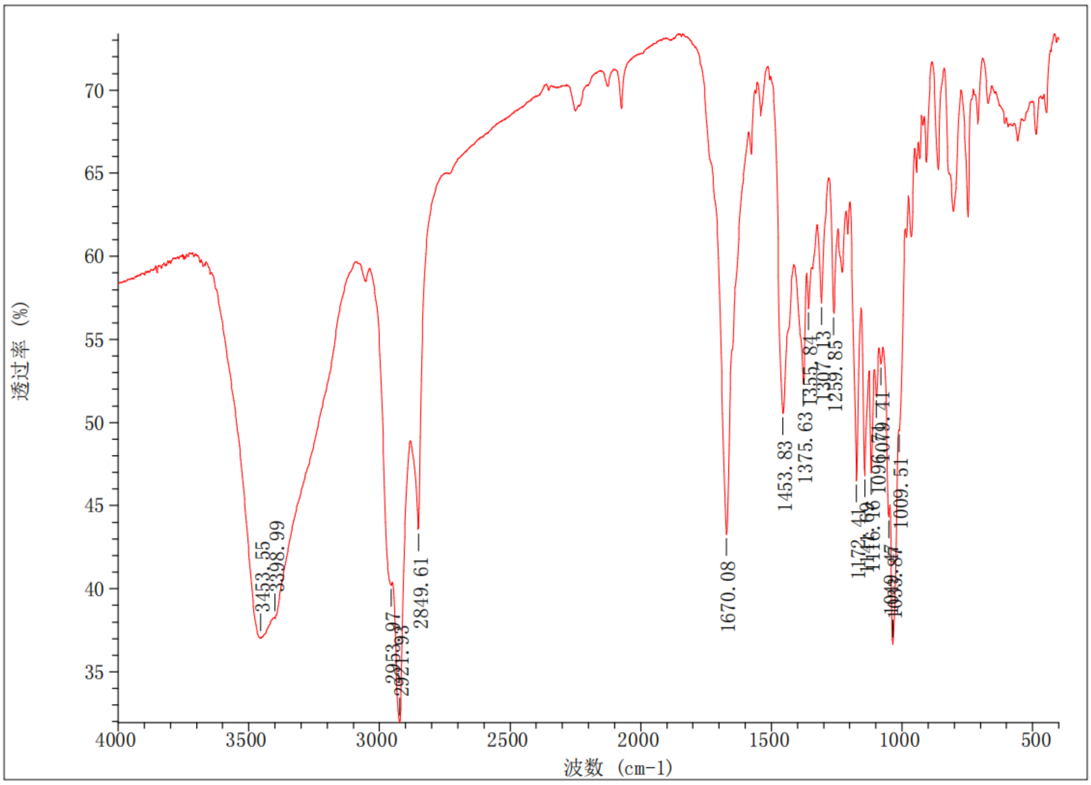

Figure S32. FT-IR spectrum of compound **2**.

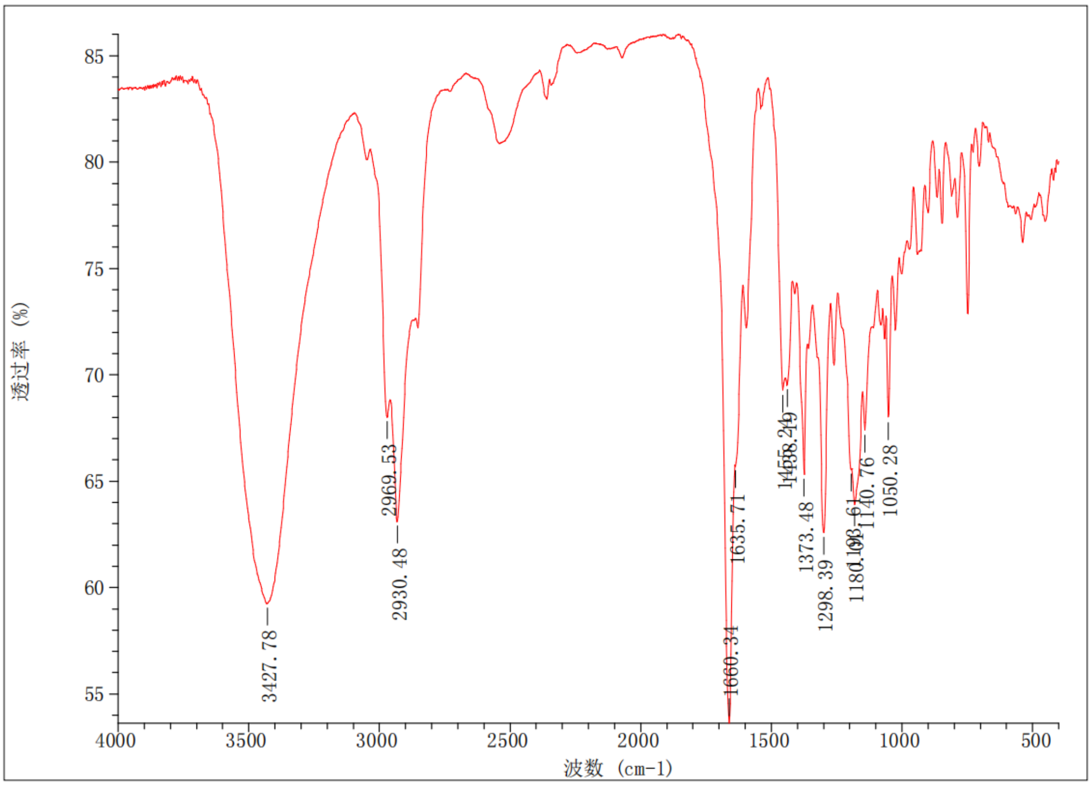

Figure S33. FT-IR spectrum of compound **3**.

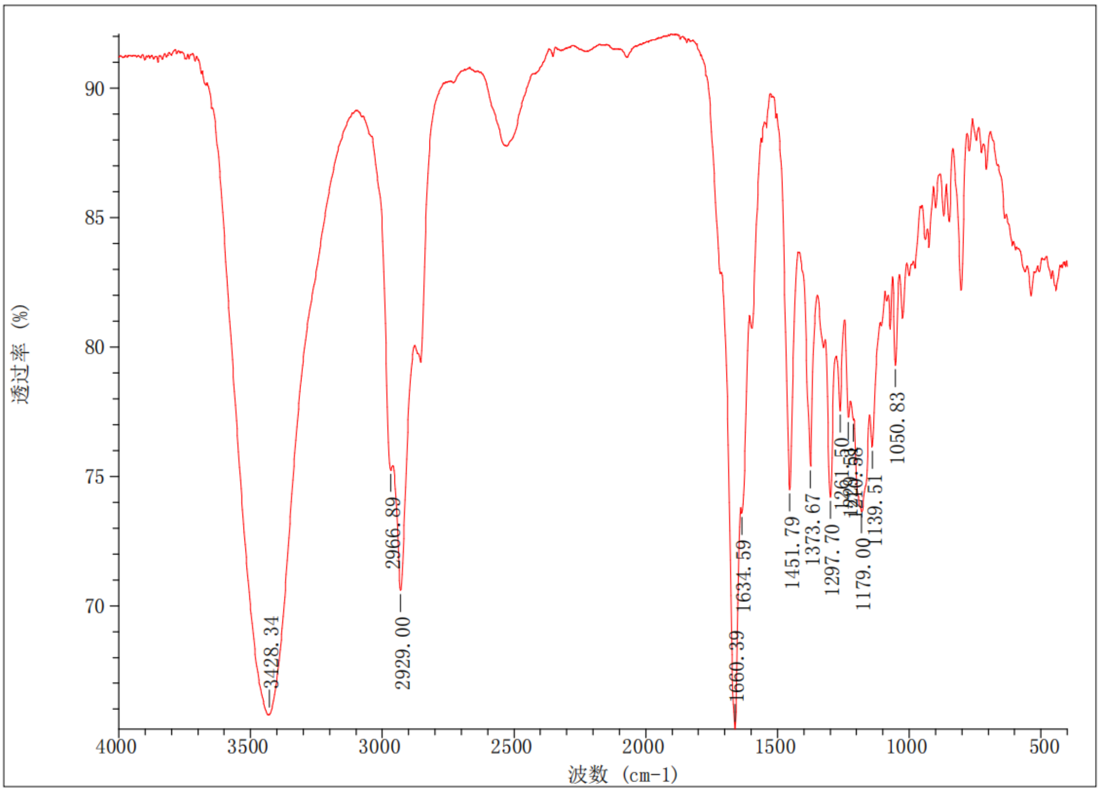

Figure S34. FT-IR spectrum of compound **4**.
